# Strategies to Control Human Health Risks Arising from Antibiotics in the Environment: Molecular Modification of QNs for Enhanced Plant–Microbial Synergistic Degradation

**DOI:** 10.3390/ijerph182010610

**Published:** 2021-10-10

**Authors:** Peixuan Sun, Wenjin Zhao

**Affiliations:** College of New Energy and Environment, Jilin University, Changchun 130012, China; sunpx19@mails.jlu.edu.cn

**Keywords:** quinolones, plant–microbial synergistic degradation, transformation pathways, 3D-QSAR, molecular dynamics, pharmacokinetics, toxicokinetics, human health risk assessment

## Abstract

In the present work, a comprehensive screening and evaluation system was established to improve the plant–microbial synergistic degradation effects of QNs. The study included the construction of a 3D-QSAR model, the molecular modification, environmental friendliness and functional evaluation of drugs, degradation pathway simulation, and human health risk assessment. Molecular dynamics was applied to quantify the binding capacity of QNs toward the plant degradation enzyme (peroxidase) and microbial degradation enzymes (manganese peroxidase, lignin peroxidase, and laccase). The fuzzy comprehensive evaluation method was used in combination with the weighted average method for normalization and assigning equal weights to the plant and microbial degradation effect values of the QNs. Considering the synergistic degradation effect value as the dependent variable and the molecular information of the QNs as the independent variable, a 3D-QSAR model was constructed for the plant–microbial synergistic degradation effect of QNs. The constructed model was then employed to conduct the molecular modification, environmental friendliness and functional evaluation, degradation pathway simulation, and human health risk assessment of transformation products using pharmacokinetics and toxicokinetics. The results revealed that the synergistic degradation effect 3D-QSAR (CoMSIA) model exhibited good internal and external prediction ability, fitting ability, stability, and no overfitting phenomenon. Norfloxacin (NOR) was used as the target molecule in the molecular modification. A total of 35 NOR derivatives with enhanced plant–microbial synergistic degradation effect (1.32–21.51%) were designed by introducing small-volume, strongly electronegative, and hydrophobic hydrogen bond receptor groups into the active group of the norfloxacin structure. The environment-friendliness and the functionality of NOR were evaluated prior to and after the modification, which revealed seven environment-friendly FQs derivatives exhibiting moderate improvement in stability and bactericidal efficacy. The simulation of the NOR plant and microbial degradation pathways prior to and after the modification and the calculation of the reaction energy barrier revealed Pathway A (D-17 to D-17-2) and Pathway B (D-17 to D-17-4) as the most prone degradation pathways in plants and Pathway A (D-17 to D-17-1) and Pathway B (D-17 to D-17-4) as the most prone degradation pathways in microorganisms. This demonstrated that the degradation of the modified NOR derivatives was significantly enhanced, with the hydroxylation and piperazine ring substitution reaction playing an important role in the degradation process. Finally, the parameters, including hepatotoxicity, mutagenicity, and rodent carcinogenicity, among others, predicted using the pharmacokinetics and toxicokinetics analyses revealed a significant reduction in the human health risk associated with the modified NOR, along with a considerable reduction in the toxicity of its transformation products, implying that the human health risk associated with the transformation products was reduced remarkably. The present study provides a theoretical basis for novel ideas and evaluation programs for improving the plant–microbial synergistic degradation of the QNs antibiotics for source control and drug design, thereby reducing the residues of these antibiotics and the associated hazard in the complex plant–soil environment, ultimately decreasing the potential risks to human health.

## 1. Introduction

The consumption of quinolones (QNs) antibiotics in China is high and accounts for approximately 15% of the total consumption of antibiotics consumed by humans, livestock, and poultry [1,2]. QNs have a long half-life, therefore, do not degrade easily and are not absorbed completely by organisms [3,4]. Approximately 30–90% of the QNs antibiotics are discharged from the body of organisms through feces or urine into the soil, plants, and water bodies, thereby causing pollution, reaching humans via the food chain, endangering human health, and reducing the effectiveness of treatment [5,6]. According to reports, urban sewage treatment plants have a low rate of removal of QNs [7,8,9], with microbial degradation and sediment adsorption being the main methods employed for QNs removal [10,11,12,13]. Alexy et al. studied the degradation ability of 18 different antibiotics and observed that the ofloxacin (OFL) removal rate was only 7.5% [14]. Senta et al. studied antibiotics removal in the urban sewage treatment plants in Croatia and reported that fluoroquinolones (FQs) exhibited strong adsorption and a low biological removal rate (8–22%) when solid particles were used [15]. Rodriguez-Mozaz et al. evaluated the concentration of FQs in wastewater from hospitals and municipalities, and the effluent discharged into rivers from sewage treatment plants [16]. The authors reported that in downstream rivers, the concentration of OFL was as high as 131 ng/L and the concentration of ciprofloxacin (CIP) was 10 times higher than that in upstream rivers, which revealed the low FQs removal efficiency of these sewage treatment plants. Xiong et al. reported that the overall levofloxacin (LEV) removal rate in wastewater treatment plants was less than 10% [17]. Zhang et al. evaluated the concentration and removal rate of antibiotics in 12 municipal sewage treatment plants in Dalian and reported that the average FQs removal rate in certain sewage treatment plants was 20.3%, while the macrolides (MLs) removal rate was as high as 90.1% [18]. Moreover, QNs exert a strong binding force on soil particles [19]. In addition, the ability of soil microorganisms to remove QNs is also quite limited, which results in the persistence of QNs residues in the soil environment [20]. For instance, Chen et al. reported that over 80% of the soil microorganisms could not degrade danofloxacin (DAN) [21].

Numerous studies have demonstrated that the rhizosphere effect is capable of promoting the degradation of antibiotics to a certain extent [22]. The rhizosphere effect alters the physical and chemical properties of the soil [23,24], the soil microorganism community [25], nutrient uptake by roots, and the root exudate release [26,27], thereby remarkably affecting the removal and subtraction of antibiotics present in the soil. Plants and rhizosphere soil microorganisms work in synergy to affect the removal of antibiotics from the soil. Chekol et al. reported that the plant rhizosphere effect significantly increased the number of soil microorganisms and the associated enzyme activities, thereby promoting the degradation of polychlorinated biphenyls (PCBs) [28]. Gilbert et al. reported that oxygen transport in plant roots and the secretion of small molecular weight organic molecules in the rhizosphere promoted the degradation of PCBs [29]. Yi et al. compared the root exudates from 43 plants in terms of their effects on the degradation of polycyclic aromatic hydrocarbons (PAHs) and reported that the pyrene degradation efficiency was different for the root exudates from different plants due to the differences in composition and nature [30]. Therefore, it is clear that the synergistic effect of plants and soil microorganisms greatly influences the degradation of QNs and the other antibiotics in the complex plant–soil environment.

Studies have demonstrated that some common degradation enzymes play key roles in the degradation of antibiotics like QNs by plant and soil microorganisms. After absorption by plants, the QNs antibiotics are degraded by peroxidase, an enzyme distributed widely in both plants and animals [31,32]. The microorganisms present in the soil may also degrade the QNs antibiotics and generate a series of degradation products [33]. For instance, white-rot fungi [34] and *Phychaete chrysosporium* [35] mineralize the pollutants by using non-specific enzyme systems, including extracellular lignin-modified enzymes (manganese peroxidase, laccase, and lignin peroxidase, etc.) and intracellular enzymes (cytochrome P450 system) [36]. Horseradish peroxidase (HRP) was reported to effectively remove sulfamazine (SMR) in a relatively short time, exhibiting a removal rate of 79.7% [37]. Potato pulp peroxidase reportedly removed 98% of 2,4-dichlorophenol (2,4-DCP) under the most favorable reaction conditions [38]. The manganese peroxidase from *Trametes versicolor* exhibited complete removal of NOR, CIP, and OFL within 14 days [39]. The removal rate of NOR by laccase and the P450 enzyme from *Phanerochaete chrysosporium* may reach 90% within seven days [35].

Furthermore, antibiotics could exert toxic effects on soil organisms, terrestrial animals, and plants, damage the skin and intestinal health of earthworms and other organisms [40], inhibit photosynthesis in plants, and destroy the cellular structures and tissue function [41,42]. Moreover, antibiotics may induce a large number of drug-resistant pathogenic bacteria and lead to the issue of antibiotic resistance gene (ARG) [43], thereby raising a greater threat to the environment, animals and plants, and human health by reducing the ability to prevent and control diseases [44,45]. In addition, the antibiotics would alter the community structure of soil microorganisms [46], thereby affecting the growth and development of plants [47,48]. Most antibiotics remain active even after being metabolized inside the bodies of organisms and might even be further converted to toxic products [49,50]. The current assessment of the risks to human health due to the ingestion of QNs contaminants through diet, inhalation, dermal contact, etc., is inadequate, with the risk levels greatly underestimated [51]. Therefore, studies investigating the residues and transformation of QNs as emerging pollutants in soil and plants and deciphering their potential risks to the ecological environment and human health have become a focal point in the research field in China as well as across the world.

On this basis, the present study was aimed to establish a comprehensive screening system based on 3D-QSAR model construction, molecular modification, drug environmental friendliness and functional evaluation, degradation pathway simulation, and human health risk assessment for application to improve the plant–microbial synergistic degradation effect of QNs. The study commenced with the use of molecular dynamics (MD) to quantify the binding capacity of QNs toward the common degradation enzymes present in different plants and microorganisms. Next, the fuzzy comprehensive evaluation method was used in combination with the weighted average method for the normalization and assigning equal weights to the plant and microbial degradation effect values of QNs. Subsequently, using the synergistic degradation effect value and the molecular information of QNs as the dependent and independent variable, a three-dimensional quantitative structure–activity relationship (3D-QSAR) model was constructed for the plant–microbial synergistic degradation effect of QNs. Using the 3D contour map of the constructed model, norfloxacin (NOR) was used as the target molecule for the subsequent molecular modification and screening of the environment-friendly derivatives of QNs exhibiting high synergistic degradation. Finally, the microbial and plant degradation pathways of NOR prior to and after the molecular modification were simulated, the energy barrier (ΔE) was calculated, and the human health risk of the transformation products was assessed using pharmacokinetics and toxicokinetics, which provide the theoretical basis for novel ideas for source control, drug design, risk assessment, and other fields associated with the QNs antibiotics.

## 2. Materials and Methods

### 2.1. Data Source of the Plant–Microbial Synergistic Degradation Effect of QNs

**Figure 1:**
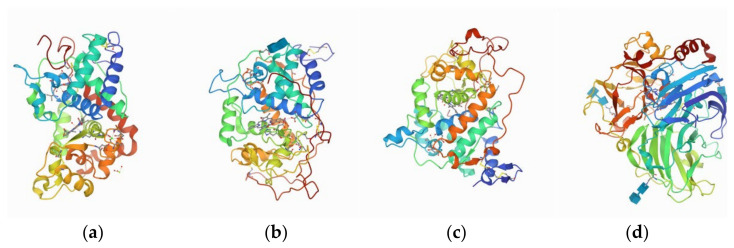
The structures of (**a**) 1PA2; **(b**) 1MNP; (**c**) 1B85; and (**d**) 1GYC enzymes.

The Protein Data Bank (PDB) database (http://www.rcsb.org/pdb (accessed on 20 October 1971)) is a recognized basic repository for free access to the three-dimensional structures of most protein, DNA, RNA, and related compounds [52]. In the present study, the structures of four common degradation enzymes present in plants and microorganisms (Figure 1), namely, peroxidase (POD, PDB ID: 1PA2) [53] from *Arabidopsis thaliana*, manganese peroxidase (MnP, PDB ID: 1MNP) [54], and lignin peroxidase (LiP, PDB ID: 1B85) [55] from the white-rot basidiomycete *Phanerochaete chrysosporium*, and laccase from white-rot fungi (Lac, PDB ID: 1GYC) [56] were searched from the PDB database.

The molecular docking method was employed to dock the QNs ligands with the above protein receptors under the Dock Ligands module of Discovery Studio (DS) (BIOVIA Inc., Shenzhen, China) software [57]. The proteins were defined under the LibDock module, the binding sites in the receptors were obtained by the Find Sites From Receptor Cavities under the Define module, and a sphere with a radius of 9 was defined at the binding site by using Define Sphere from Selection under the Define module. Furthermore, the Docking Preferences and the Max Hits to Save were set as User Specified and 10, respectively, in the Dock Ligands module. Finally, the ligands were integrated into the formed binding cavity of the proteins for rapid docking with the receptors, obtaining the complexes of the ligand molecules and protein receptors [58,59,60].

The degree of binding of each of four degradation enzyme structures with the QNs molecules was calculated based on molecular dynamics using the Gromacs 4.6.5 software (Karolinska Institutet, Stockholm University, Stockholm, The Kingdom of Sweden) of the Dell PowerEdge R7425 server [61,62,63]. The complex system of QNs molecules and enzymes was placed inside a 12-periodic cube with a side length of 15 nm. The GROMOS96–43a1 force field was applied for molecule restriction, and Na^+^ was added to neutralize the system charge, rendering the whole system electrically neutral. The steepest gradient method was adopted for energy minimization simulation, and the number of simulation steps was set at 5,000,000 for 1-ns simulation. In the canonical ensemble (NVT) and the constant-pressure and constant-temperature ensemble (NPT) simulation, the temperature was set to the indoor temperature (300 K) [64], and the size of the pressure bath was set to a constant standard atmospheric pressure (1 bar). The binding energy (G_bind_, ΔG_b_, kcal/mol) was analyzed using Molecular Mechanics Poisson–Boltzmann Surface Area (MMPBSA) [65], quantifying the binding degree between the QNs molecules and each degradation enzyme, and finally expressing the binding capacity of the ligands and receptors in terms of binding energy values, i.e., the plant–microbial synergistic biodegradability, which was used as the data basis for the study on the plant–microbial synergistic degradation effect of QNs in the present study. The smaller the binding energy values (usually negative) and the larger the absolute values, the stronger is the binding capacity between the degradation enzymes and the QNs molecules, which indicates a greater plant–microbial synergistic degradation effect of QNs.

### 2.2. D-QSAR Model of the Plant–Microbial Synergistic Degradation Effect of QNs

#### 2.2.1. Characterization of the Plant–Microbial Synergistic Degradation Effect of QNs—Fuzzy Comprehensive Evaluation Method

The fuzzy comprehensive evaluation method was originally proposed by Wang, which was applied widely to solve fuzzy problems difficultly to be quantified [66]. In the present study, the fuzzy comprehensive evaluation method was used in combination with the weighted average method to conduct the relative normalization treatment of the plant and microbial degradation effect values of QNs [67]. The synergistic degradation effect values of QNs were calculated using the weight ratio of 25:25:25:25% and subsequently utilized to characterize the plant–microbial synergistic degradability of QNs. The plant–microbial synergistic degradability of the QNs molecules was evaluated, revealing the factor set *U* comprising four degradation enzymes (which were selected as the main factors of the evaluated objects), while the values of binding energy between the QNs molecules and each degradation enzyme constituted the judgment set *V*_j_. The expressions for these two sets are provided below.

*U* = { u_1_, u_2_, …, u_j_, …, u_m_}   m = 4 (1)

*V*_j_ = {v_1_, v_2_, …, v_i_, …, v_n_} j = 1, …, 4, n = 29 (2)

where u_j_ represents the j-th evaluation factor, i.e., the j-th degradation enzyme, *V*_j_ represents the numerical set of the binding energies between the j-th degradation enzyme and the QNs molecules, v_i_ represents the value of the single-effect binding energy corresponding to the i-th molecule, m is the number of degradation enzymes, and n is the number of QNs molecules.

A fuzzy comprehensive evaluation matrix *R* comprising the values of binding energy between the QNs molecules and each of the four enzymes was constructed. The weight vector *W* for each of the four degradation enzymes was determined using the weighted average method. The expressions for *R* and *W* are provided below.

(3) $$r=\left[\begin{array}{cccc} r_{11} & r_{12} & \ldots & r_{1n}\\ r_{21} & r_{22} & \ldots & r_{2n}\\ \vdots & \vdots & r_{\mathrm{ij}} & \vdots \\ r_{m1} & r_{m2} & \ldots & r_{\mathrm{mn}} \end{array}\right]\;m\;=\;29,\;n\;=\;4$$

*W* = (a_1_, a_2_, a_3_, a_4_) (4)

where r_ij_ represents the value of binding energy between the i-th molecule and the j-th degradation enzyme. All four degradation enzymes were assigned equal weights.

The application of the weighted average fuzzy operator o on the weight vector *W* and matrix *R* generated the fuzzy vector *B*, and the molecular plant–microbial synergistic degradation effect values of QNs denoted by b_i_. The corresponding expressions are provided below.


(5) $$\circ :M(•,⊕):b_{m}=\min(1,\;\sum_{i=1}^{4}\;a_{i}r_{\mathrm{mn}})\;m\;=\;1,\;2,\;\ldots ,\;29\;,\;n\;=\;1,\;\ldots ,\;4$$

(6) $$B=W\;\circ \;R=(\;a_{1},\;a_{2},\;a_{3},\;a_{4})\;\left[\begin{array}{cccc} r_{11} & r_{12} & \ldots & r_{1n}\\ r_{21} & r_{22} & \ldots & r_{2n}\\ \vdots & \vdots & r_{\mathrm{ij}} & \vdots \\ r_{m1} & r_{m2} & \ldots & r_{\mathrm{mn}} \end{array}\right]\;=\;(b_{1},\;b_{2},\;\ldots ,b_{i},\;\ldots ,b_{m})$$

#### 2.2.2. Construction of the 3D-QSAR Model for the Plant–Microbial Synergistic Degradation Effect of QNs

**Figure 2:**
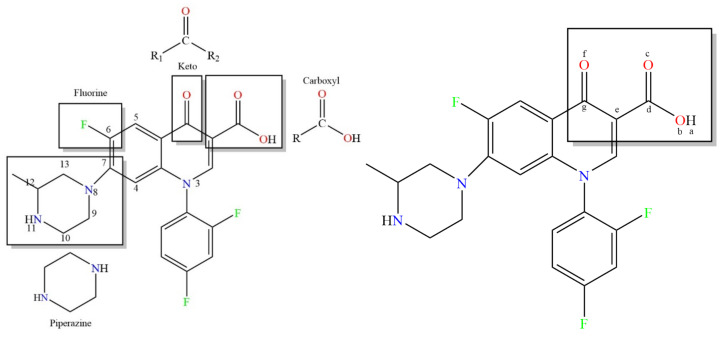
The molecular structure and the common skeleton of TEM. “a–f” indicate atoms H, O, O, C, O, and C, respectively, in the common skeleton.

In the present study, a modified three-dimensional quantitative structure–activity relationships (3D-QSAR) model of plant–microbial synergistic degradation effect was constructed by combining the fuzzy comprehensive evaluation method and the traditional QSAR method and comprehensively considering the four degradation enzymes and dual effects. The Sybyl-X2.0 (Tripos Inc., Saint Louis, MO, USA) software was used to perform the 3D-QSAR analysis and to construct the comparative molecular similarity indices analysis (CoMSIA) model for the plant–microbial synergistic degradation effect of QNs. First, the structures of the QNs molecules were obtained using the Sketch Molecule module of Sybyl-X2.0. Since the molecular structures constructed did not represent the most stable conformations of the respective molecules, the conformation with the lowest energy was generally selected for each molecule in the case of an unknown receptor. In the software, the Powell conjugate gradient method under the Minimize module was adopted, the Tripos force field was selected, the Gasteiger-Hückel charge was added, energy convergence was limited to 0.005 kJ/mol after 10,000 iterations, and the other parameters were set to default values for molecular optimization [68]. Temafloxacin (TEM), a third-generation fluoroquinolone antimicrobial agent exhibiting the largest synergistic degradation effect, was selected as the template molecule. The common structure of the QNs molecules was selected as the common skeleton (Figure 2). The Align Database module was used to perform the skeleton alignment of the QNs molecules. The optimized molecules were allocated to the training and test sets randomly, and both training and test sets contained the template molecule.

**Figure 3:**
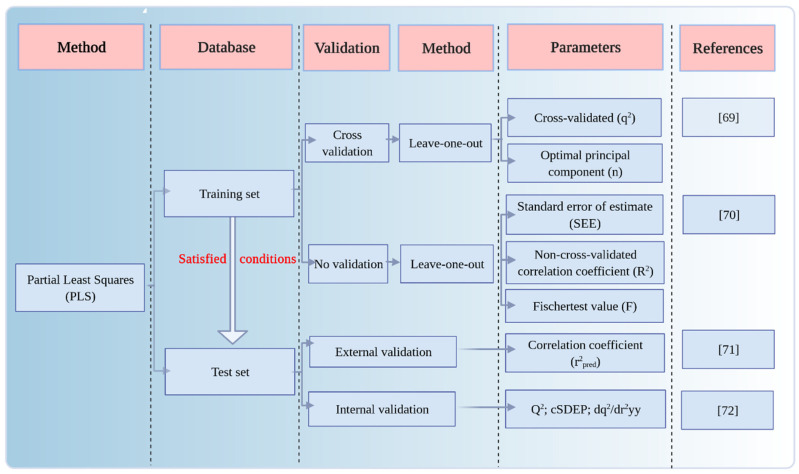
The specific method and the associated parameters of model construction (created with https://biorender.com (accessed on 9 October 2017)).

When calculating the parameters of the constructed CoMSIA model, the molecular field types, namely the steric field (S), electrostatic field (E), hydrophobic field (H), hydrogen bond acceptor field (A), and hydrogen bond donor field (D), were applied to elucidate the structure–activity relationship of the compounds directly, and led to the construction of the CoMSIA model for the plant–microbial synergistic degradation effect and the single effect of QNs, respectively (Figure 3).

### 2.3. Evaluation of the Environment-Friendliness and Functional Properties of the QNs Derivatives—EPI, Gaussian, Pharmacodynamics, and HQSAR Model

In the present study, bioaccumulation (log *K*_ow_) and soil adsorbability (log *K*_oc_) were used as indices for the evaluation of the environment-friendliness of QN derivatives. The EPIWEB 4.1 (Estimation Programs Interface) software (OTTP of EPA&SRC, Washington, DC, USA) was employed to predict the log *K*_ow_ and log *K*_oc_ values for the QNs derivatives. Furthermore, stability (molecular structure stability as well as molecular metabolic stability) and genotoxicity were used as indices for the functional evaluation of QNs derivatives to ultimately determine the degree of improvement in the molecular function of NOR derivatives. The molecular structure stability was defined based on the positive frequency (cm^−1^) and the total energy (a.u.) at the unit level of b3pw91/6-31G*, according to the density functional theory (DFT), calculated using the GAUSSVIEW 5.0 software (Gaussian Inc., Wallingford, CT, USA) [69,70,71]. In this software, the GIF format molecules were loaded, optimized, and finally output the result files. The molecular metabolic stability of the QNs derivatives in the human body was predicted using an evaluation model for the combination of the human cytochrome P450 2D6 enzyme (CYP450 2D6) and the molecules under the ADMET module in DS software [72]. The ADMET Descriptors of Calculation Molecular Properties under the ADMET module were used to predict the Bayesian scores which represented the pharmacokinetics properties of QNs and derivatives. In addition, the negative logarithm of the Lowest Observed Effect Concentration (pLOEC) was predicted using the HQSAR model for the genotoxicity of quinolones toward *Salmonella typhimurium*, constructed by Zhao et al., to evaluate the bactericidal effect of NOR and its derivatives [73].

### 2.4. Simulation of the Plant and Microbial Transformation Pathways of QNs and Their Derivatives

**Figure 4:**
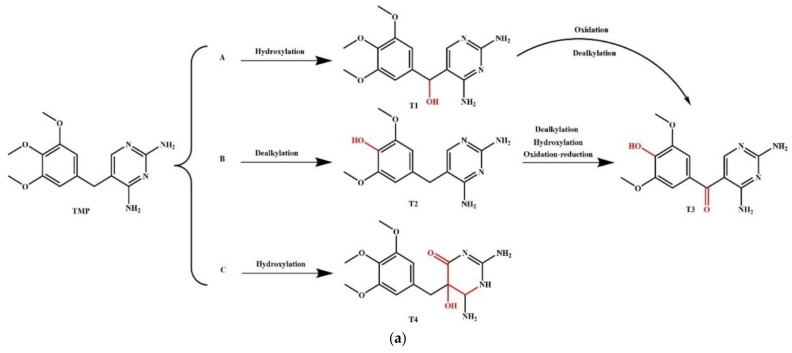

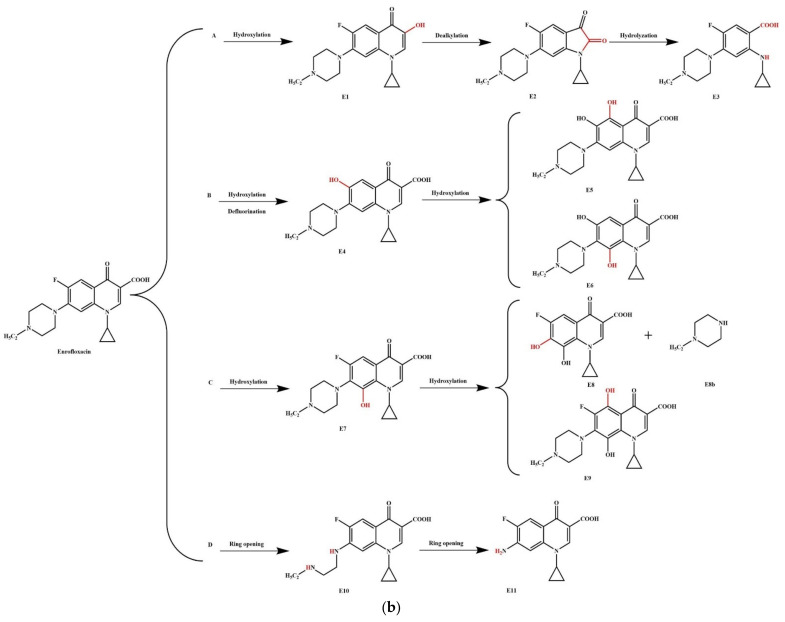
Simulation of (**a**) plant; and (**b**) microbial degradation pathways of antibiotics in previous studies.

The intermediates and the final products of plant and microbial transformation of QNs and their derivatives were identified based on the main pathways of trimethoprim (TMP) in leafy vegetables reported by Tian et al. [74] (Figure 4a) and the main pathways which the brown-rot fungi used to degrade enrofloxacin (ENR) [75] (Figure 4b). DFT and the GAUSSVIEW 5.0 software of GAUSSIAN 09 package were used for optimizing the structures of molecules, and calculating the reaction energy barrier (ΔE) and the simple harmonic vibration frequencies of the substances at the unit level of b3lyp/6-31G* [76], respectively, in order to analyze the plant and microbial transformation pathways of QNs and their derivatives. The transition state had only one virtual frequency, but the intermediate did not. The Intrinsic Reaction Coordinate (IRC) verification of the transition states was carried out [77]. Firstly, the specific parameters of the Gaussian input file (.gif) of compounds were edited. “% mem = 3000 MW” and “% nproc = 16” represented 3000 MW of memory space and 16 CPU cores for structure optimization and frequency calculation, respectively. “# b3lyp/6-31g (d, p) opt freq” was the unit level of b3lyp/6-31G* of DFT, and “iop (5/13 = 1)” represented the number of iterations which increased when the calculation results did not converge. Secondly, the edited Gaussian input files were submitted into the software and the “g09&” command was used to start optimization and betrothal calculation. Finally, the Gaussian output file (.log) was generated [78].

### 2.5. Assessment of Human Health Risk Raised by the Plant and Microbial Transformation Products of QNs and Their Derivatives Using Pharmacokinetics and Toxicokinetics

The hepatotoxicity levels of the plant and microbial transformation products of QNs and their derivatives were predicted based on pharmacokinetics using the ADMET module of the DS software (BIOVIA Inc., Shenzhen, China) [79,80]. In addition, the potential risks to human health raised by these transformation products were predicted based on toxicokinetics using the 10 toxicity models in the TOPKAT module of the DS software [81], including the developmental toxicity potential (DTP), skin sensitization, skin irritancy, ocular irritancy, mutagenicity (Ames Test), rodent carcinogenicity (NTP and FDA datasets), and rat oral toxicity (*LD*_50_), etc.

## 3. Results and Discussion

### 3.1. Construction and Evaluation of the 3D-QSAR Model for the Plant–Microbial Synergistic Degradation Effects of QNs

#### 3.1.1. Calculation of the Plant–Microbial Synergistic Degradation Effect Values of QNs

The values of the binding energy between the QNs and one plant degradation enzyme (POD, PDB ID: 1PA2) and three microbial degradation enzymes (MnP, PDB ID: 1MNP; LiP, PDB ID: 1B85 and Lac, PDB ID: 1GYC) were calculated based on molecular dynamics, followed by the relative normalization of the calculated values using the fuzzy comprehensive evaluation method in combination with the weighted average method. The weight ratio used was 25:25:25:25%. Table 1 summarizes the calculated binding energy values for the plant and microbial degradation effects of QNs along with the synergistic degradation effect values after normalization. Smaller synergistic degradation effect values (larger absolute values) obtained for the molecules indicated a stronger synergistic degradation effect.

#### 3.1.2. Construction and Evaluation of the 3D-QSAR Model for the Plant–Microbial Synergistic Degradation Effects of QNs

An effective CoMSIA model for the plant–microbial synergistic degradation effects of QNs was constructed employing Sybyl-X2.0 software. The partial least squares (PLS) module of Sybyl-X2.0 software (Tripos Inc., Saint Louis, MO, USA) was used for analysis. The cross-validated correlation coefficient (q^2^) of the constructed model was 0.707 (>0.5), and the optimal principle number of components (*n*) was eight, indicating that this model revealed a great predictive ability [82]. The non-cross-validated correlation coefficient (R^2^) of this model was 0.999 (>0.9), the standard error of estimation (SEE) was 0.308 (<0.95), and the Fischer’s test value (F) was 1156.013, indicating that this model exhibited reliable fitting ability and internal predictive ability [83]. The (R^2^ − q^2^)/R^2^ value (<30%) of the constructed model indicated that no overfitting phenomenon occurred when using this model [84]. The values of the parameters Q^2^, cSDEP, and dq^2^/dr^2^yy obtained in the perturbation stability test of the model were 0.416, 7.495, and 1.597, respectively, which indicated the good predictive ability and stability of this model [85]. Furthermore, the external validation of the model’s testing set revealed a correlation coefficient r^2^_pred_ of 0.901 (>0.6), which indicated the good external predictive ability of the model [86] (Table 2).

#### 3.1.3. Validation of the 3D-QSAR Model for the Plant–Microbial Synergistic Degradation Effects of QNs

**Figure 5:**
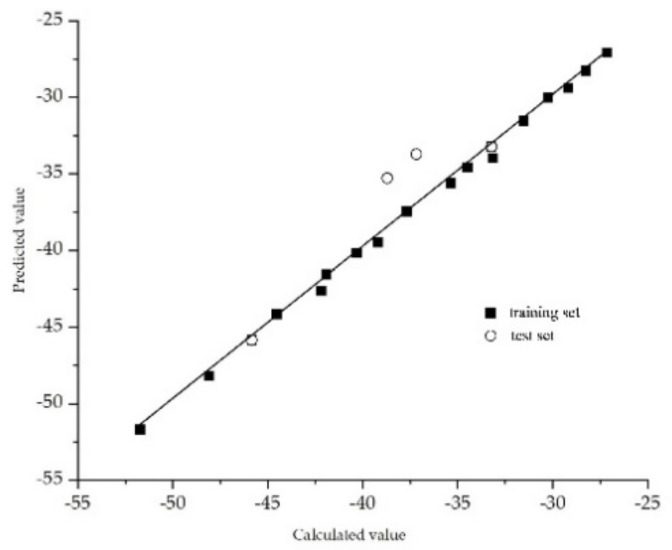
Correlation of the calculated and predicted values of plant–microbial synergistic degradation effects of QNs in the CoMSIA model.

The constructed CoMSIA model was used to predict the activity of the molecules in the training set and the test set. The accuracy of the model was tested by a linear analysis of the predicted values and the calculated values of plant–microbial synergistic degradation effects in the model. As shown in Figure 5, the predicted values in the CoMSIA model presented a linear correlation with the calculated values, and the slope of the linear equations for the calculated and predicted values was 0.988, revealing that the constructed model had good internal predictive ability and could be used to predict the plant–microbial synergistic degradation effects of QNs derivatives [68].

### 3.2. Molecular Modification of the QNs Derivatives Based on the CoMSIA Model for the Plant–Microbial Synergistic Degradation Effects

**Figure 6:**
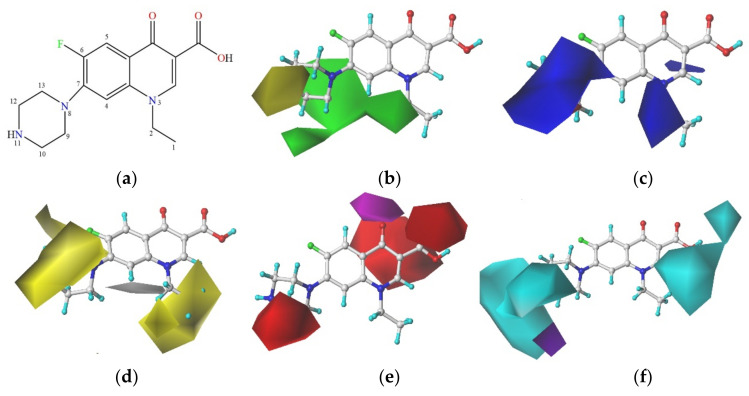
Molecular structure of NOR and the 3D contour map of the CoMSIA model: (**a**) Molecular structure of NOR; (**b**) Steric field; (**c**) Electrostatic field; (**d**) Hydrophobic field; (**e**) Hydrogen bond acceptor field; (**f**) Hydrogen bond donor field.

In the present study, NOR, which is a widely-used third-generation fluoroquinolone antibacterial agent, was selected as the target molecule for the 3D contour map analysis of the constructed CoMSIA model. The distribution principle of the color blocks in the 3D contour map revealed that the activity of the compound could be increased by increasing the group volume close to the green blocks and reducing the group volume close to the yellow blocks of the steric field and also by increasing the group electronegativity close to the blue blocks and the group electronegativity close to the red blocks of the electrostatic field. In addition, the introduction of hydrophobic groups close to the yellow blocks and hydrophilic groups close to the white blocks of the hydrophobic field could be beneficial to increasing the activity of the compound. Furthermore, the introduction of hydrogen bond acceptors close to the purple blocks and hydrogen bond donors close to the red blocks of the hydrogen bond acceptor field, as well as the introduction of hydrogen bond donors close to the cyan blocks and hydrogen bond acceptors close to the purple blocks of the hydrogen bond acceptor field could all be beneficial to increasing the activity of the compound [87]. In the present study, the binding energy parameter was used for characterizing the synergistic degradation effect of the QNs molecules; the synergistic degradation effect increased when the binding energy was reduced. Therefore, to reduce the binding energy of the compounds and enhance the synergistic degradation effect, the molecular modification was conducted in a manner contrary to the above substitution law. The molecular structure of NOR and the steric, electrostatic, hydrophobic, hydrogen bond acceptor, and hydrogen bond donor fields of the CoMSIA model are depicted in Figure 6.

As depicted in Figure 6, the contribution rate of the S, E, H, A, and D fields for the constructed model was 19.1%, 21.7%, 27.4%, 9.3%, and 22.5%, respectively. These results indicated that the electrostatic, hydrophobic, and hydrogen bond acceptor fields were the main factors affecting the plant–microbial synergistic degradation of QNs, while the effect of the hydrogen bond donor field was relatively small, and it could, therefore, be regarded as a secondary factor. As visible in Figure 6b, the green blocks are widely distributed around the –CH_3_ group at site C_1_ and the –CH_2_– group at site C_2_. Figure 6c,d revealed that the blue and white blocks are mainly distributed close to sites C_2_ and C_13_, respectively. As depicted in Figure 6e,f, both red and cyan blocks are distributed around site C_13_. In conclusion, a small group (–Br) was introduced at site C_1_, seven small groups with greater electronegativity (–F, –CH_3_, –NH_2_, –SH, –COOH, –CF_3_, and –CH_2_F) were introduced at site C_2_, three hydrophobic hydrogen bond receptor groups (–SH, –Cl, and –F) were introduced at site C_13_, and a total of 35 NOR derivatives with enhanced plant–microbial synergistic degradation effect were designed and are presented in Table 3.

**Figure 7:**
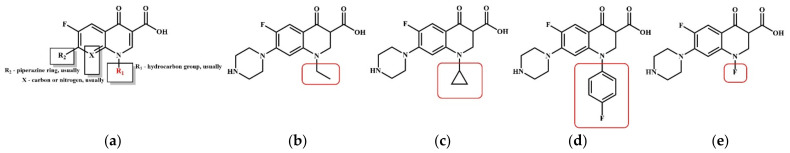
Molecular structure of FQs and derivative D-5: (**a**) Maternal structure of FQs; (**b**) Norfloxacin (NOR); (**c**) Ciprofloxacin (CIP); (**d**) Sarafloxacin (SAR); (**e**) Derivative D-5.

The molecular structure and synergistic degradation of the ciprofloxacin (CIP) (Figure 7c) and sarafloxacin (SAR) (Figure 7d), which were widely used, the target molecule norfloxacin (NOR) (Figure 7b), and the derivative D-5 (Figure 7e) were compared as examples to verify the rationality of the above substitution law. By analyzing the 3D contour map of NOR in the constructed model (Figure 6) and the molecular structure of CIP and SAR (Figure 7c,d), it was found that there were differences in the R_1_ substitution group of NOR, CIP, and SAR. The green blocks of the steric field and white blocks of the hydrophobic field were widely distributed around the R_1_ substitution group, that is, reducing the volume or increasing the hydrophobicity of the R_1_ group could improve the plant–microbial synergistic degradation of QNs molecules. In the present study, the R_1_ substitution groups of NOR and its derivative D-5 were ethyl (–C_2_H_5_) and fluorine (–F), respectively. The volume of ethyl was larger than that of fluorine, and the hydrophobicity of ethyl was smaller than that of fluorine, while the comprehensive value of NOR (−34.133 kcal/mol) was larger than the predicted value of D-5 (−39.659 kcal/mol), indicating that the synergistic degradation effect of QNs was improved after modification. The R_1_ substitution groups of NOR and CIP were ethyl and cyclopropyl, respectively (the volume of ethyl was smaller than that of cyclopropyl), and the comprehensive value of NOR (−34.133 kcal/mol) was smaller than that of CIP (−31.535 kcal/mol) (Table 1), verifying the substitution law that the smaller the volume of R_1_ substitution group, the greater the synergistic degradation effect. Furthermore, studies have shown that hydrophilicity and hydrophobicity of groups are correlated with log P value (the larger the log P, the stronger the hydrophobicity) [88]. The R_1_ substitution groups of CIP and SAR were cyclopropyl (log P = 1.25, hydrophilic group) and fluorophenyl (log P = 2.19, hydrophobic group), respectively, and the comprehensive value of SAR (−32.816 kcal/mol) was smaller than that of CIP (−31.535 kcal/mol) (Table 1.), verifying the substitution law that the stronger the hydrophobicity of R_1_ substitution group, the greater the synergistic degradation effect. The present study had verified and analyzed the rationality of molecular modification based on 3D contour map and groups properties [89]. Therefore, the molecular modification based on the 3D contour map of the CoMSIA model, the molecular structure, and the properties of groups had rationality and reliability in the present study.

### 3.3. Prediction and Evaluation of the Plant–Microbial Synergistic Degradation Effects of the Modified NOR Derivatives

The synergistic degradation effect values and change rates for NOR derivatives predicted by the constructed CoMSIA model was visible in Table 4. The predicted values of the 35 NOR derivatives exhibited a decrease of 1.32–21.51%, i.e., the degradability by both plants and microorganisms had increased. The comprehensive predicted values for 9 NOR derivatives, namely, D-1, D-2, D-13, D-14, D-15, D-16, D-18, D-21, and D-23, exhibited a significant decrease (>18%), indicating a remarkable enhancement in the synergistic degradability. Among these, seven derivatives (D-1, D-2, D-13, D-14, D-15, D-16, and D-18) had the substitution at site C_13_, and the substituent group sulfhydryl (–SH) belonged to the group of hydrogen bond receptors with strong hydrophobicity [90,91], which confirmed that the hydrophobic and hydrogen bond receptor fields in the CoMSIA model were the main factors affecting the plant–microbial synergistic degradation effect of QNs.

### 3.4. Evaluation of the Environment-Friendliness and the Functional Properties of NOR Derivatives

#### 3.4.1. Evaluation of the Environment-Friendliness of NOR Derivatives

Studies have demonstrated that adsorption is critical to the migration of antibiotics in the soil as well as their environmental fate [92]. Antibiotic residue and accumulation in soil could result in the multiplicand of drug-resistant genes [93,94,95,96], which, when introduced into the food chain, would cause chronic adverse harm to animals, plants, as well as humans [97,98,99,100]. QNs exhibit a high degree of adsorption in soil. Pan et al. reported a high degree of soil adsorption for NOR, with the adsorption coefficient *K*_d_ of approximately 591 L/kg [101]. Golet et al. reported that QNs exhibited high persistence and limited mobility in soil [12]. Bioaccumulation of antibiotics is another measure of their environment-friendliness. Schafhauser et al. reported that the environmental concentration of erythromycin in aquatic organisms in the inland waters of Asia exceeded the ERY food safety tolerance levels established by the US Food and Drug Administration by approximately 5%, while this percentage was 7% in fish [102]. Michelini et al. reported that the high concentration of sulfadiazine in willow and maize caused severe stress to plants and even plant death in certain cases [103]. Han et al. observed that exogenous substances, such as microplastics, could amplify the bioaccumulation of veterinary drugs in Mytilus coruscus and induce synergistic immunotoxic effects [104]. Therefore, reducing the bioaccumulation and soil adsorbability of QNs is of great significance to both human beings and the environment.

In the present study, the bioaccumulation (log *K*_ow_) and soil adsorbability (log *K*_oc_) of the NOR derivatives involved were predicted (Table 5). D-5, D-15, D-17, D-21, D-23, D-24, D-25, D-29, D-31, and D-33 exhibited decreased values of log *K*_ow_ and log *K*_oc_ (in the ranges of 12.62–166.99% and 12.76–237.24%, respectively), implying that both bioaccumulation and soil adsorbability of these derivatives were reduced. However, the derivatives corresponding to the minimum and maximum change rates in these two properties remained the same (D-21 and D-33). Moreover, the change rates of the bioaccumulation and soil adsorbability of the above-stated 10 derivatives were of the same order. Among the other 16 derivatives that presented increased predicted values for bioaccumulation and soil adsorbability, although D-30 and D-35 presented slightly increased values of log *K*_ow_ and log *K*_oc_, the amplitude was small, indicating that the bioaccumulation and soil adsorbability of these two derivatives remained unchanged fundamentally.

According to the above results, 12 NOR derivatives (including D-5, D-15, D-17, D-21, D-23, D-24, D-25, D-29, D-30, D-31, D-33, and D-35) were considered environment-friendly QNs derivatives exhibiting increased synergistic degradation along with significantly decreased or fundamentally unchanged bioaccumulation and soil adsorbability.

#### 3.4.2. Evaluation of the Functional Properties of NOR Derivatives

Studies have evaluated the functional properties of derivatives of different antibiotics. Li et al. used density functional theory (DFT) to determine the molecular structure stability characterized by the positive frequency values [105]. Zhang et al. reported using molecular metabolic stability and genotoxicity as indices for the functional evaluation of FQs derivatives [106]. Both Zhang et al. and Hou et al. reported using the HQSAR model for the genotoxicity of quinolones toward Salmonella typhimurium for predicting the negative logarithm lowest observed effect concentration (pLOEC) values, which were then used for characterizing bacterial genotoxicity [59,107].

In the present study, the parameters and their change rates were obtained in the functional evaluation of NOR and its derivatives (Table 6), and the evaluation criteria are visible in Table 7. The analysis of the stability parameters revealed that the positive frequency value of each of the evaluated 12 NOR derivatives was positive, indicating that these derivatives could exist stably in the environment. The total energy values of these 12 NOR derivatives exhibited different degrees of reduction (1.85–51.30%), indicating that the environmental stability of these NOR derivatives was better than that of the original NOR molecules. The Bayesian scores for NOR as well as its derivatives were all less than 0.161 (referred to as non-inhibitor), indicating a certain degree of molecular metabolic stability in NOR and its derivatives. In addition, the genotoxicity of D-5, D-15, D-17, D-23, D-24, D-25, and D-31 remained fundamentally unchanged, indicating a certain degree of bactericidal efficacy in these derivatives. On the basis of the results of the evaluation of environment-friendliness and functional properties, the above-stated seven derivatives (D-5, D-15, D-17, D-23, D-24, D-25, and D-31) were considered environment-friendly QNs derivatives exhibiting moderately improved molecular stability and bactericidal efficacy.

### 3.5. Simulation of the Plant and Microbial Transformation Pathways of NOR and Its Derivatives

#### 3.5.1. Simulation of the Plant Degradation Pathways of NOR and Its Derivatives

**Figure 8:**
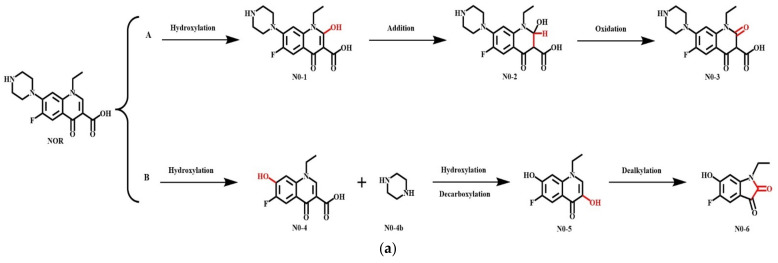

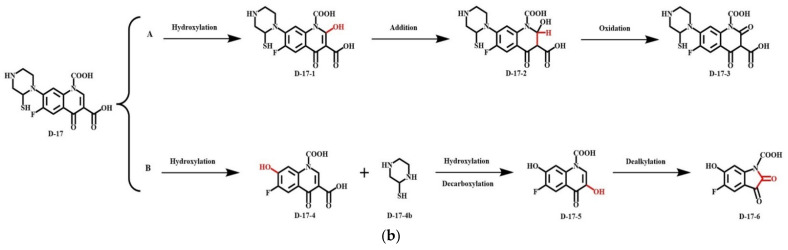
Simulation of the plant degradation pathways of (**a**) NOR; and (**b**) its derivative D-17.

Antibiotics that enter the soil environment could catalyze a series of transformations and degradations under the combined action of plants and microorganisms. After being absorbed by plant roots, these antibiotics are transported to the stems, leaves, and fruits, where these are transformed into different metabolites through the action of the related enzymes [111,112,113,114,115]. The relevant studies in the existing literature mainly focused on the residue of antibiotics in plants but lacked the analysis of antibiotic degradation, the internal mechanism of degradation, and the toxicity of the products of antibiotics after transformation in plants [116]. The studies exploring the plant degradation pathways of QNs are scarce. In the present study, two main plant degradation pathways (involving the processes of hydroxylation, decarboxylation, and dealkylation) of QNs were speculated, and the pathways of NOR and its derivative D-17 were simulated based on the previous study [78] (Figure 8). The ΔE was calculated (Table 8), and the toxicity levels of the main transformation products were predicted based on pharmacokinetics and toxicokinetics (Table 9). Moreover, the most probable degradation pathways with the lowest risk were screened for risk assessment.

As visible in Figure 8, the QNs derivative D-17 could undergo hydroxylation of the pyridoxylic acid-biphenyl moiety, generating the hydroxylated product D-17-1, which further underwent an addition reaction and bond breakage to generate the intermediate product D-17-2 that was eventually oxidized to D-17-3 (pathway A). Moreover, the piperazine ring at site C_7_ could be replaced by an –OH group to generate the hydroxylation products D-17-4 and D-17-4b, which further underwent the oxidative decarboxylation reaction. This reaction produced the intermediate product D-17-5 through the substitution of the –COOH group, while the six-membered ring was opened, then transformed into a five-membered ring under the action of –OH to finally generate the dealkylation product D-17-6 (pathway B).

#### 3.5.2. Simulation of the Microbial Degradation Pathways of NOR and Its Derivatives

**Figure 9:**
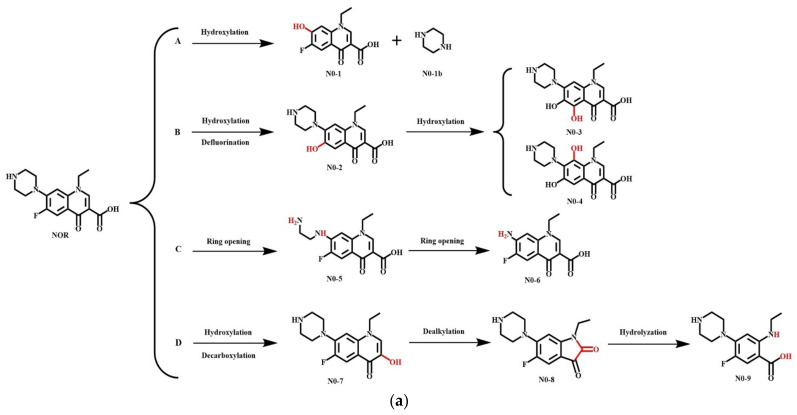

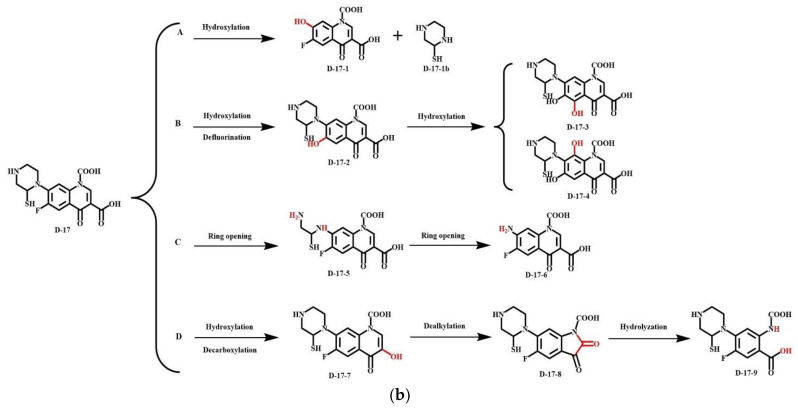
Simulation of the microbial degradation pathways of (**a**) NOR; and (**b**) its derivative D-17.

Soil microorganisms degrade the antibiotics into a series of products via four major pathways (hydroxylation, oxidative defluorination, piperazine ring pyrolysis, and oxidative decarboxylation reaction), as reported in previous studies [75,118,119]. These four pathways of NOR and its derivative D-17 were simulated in the present study (Figure 9), based on which the ΔE was calculated (Table 8), and the potential toxicity levels of the main products were predicted (Table 9). In addition, the most probable degradation pathways with the lowest risk were screened for risk assessment.

As illustrated in Figure 9, –OH played a vital role in the degradation as it replaced the piperazine rings to generate the hydroxylation products D-17-1 and D-17-1b (pathway A) [120]. In addition, the C–F bond was activated to undergo the oxidative defluorination reaction, in which F (F_6_) was replaced with –OH to generate the intermediate product D-17-2. Subsequently, the C atoms at the ortho-position (C_5_) or para-position (C_4_) were activated to finally generate the hydroxylated product D-17-3 or D-17-4 (pathway B). The piperazine ring was highly prone to the ring-opening cleavage that resulted in the generation of the semi-ring-opening intermediate product D-17-5 [120], which was unstable and ultimately transformed to the ring-opening product D-17-6 (pathway C). Moreover, the intermediate product D-17-7 could be formed in an oxidative decarboxylation reaction, followed by the opening of the six-membered ring to generate a five-membered ring under the action of –OH, thereby forming the dealkylation product D-17-8, which then underwent the hydrolysis reaction to form the decarboxylation product D-17-9 (pathway D).

It was revealed that ΔE (>0) could explain the degree of difficulty in the occurrence of the reaction. The smaller the energy barrier, the more probable was the reaction [121]. As presented in Table 8, the energy barriers of the first two steps of pathway A and the first hydroxylation reaction of pathway B of the plant degradation process were reduced by 98.61%, 98.37%, and 56.54%, respectively. This indicated a higher probability of piperazine ring replacement and oxidation reaction occurrences in the modified derivatives. During the microbial degradation, the energy barriers of the two pathways (A and B) of the modified derivative D-17 were significantly decreased (56.54% and 77.16%, respectively), indicating that the microbial capacity for the degradation of QNs was significantly improved after QN modification. Therefore, after the modification of QNs derivatives, pathway A (D-17 to D-17-2) and pathway B (D-17 to D-17-4) were revealed as the main pathways for the plant degradation of QNs, while the main pathways for the microbial degradation of QNs were pathway A (D-17 to D-17-1) and pathway B (D-17 to D-17-4). The –OH group played a significant role in both plant and microbial degradation of QNs.

### 3.6. Assessment of the Human Health Risk Raised by the Plant and Microbial Transformation Products of NOR and Its Derivatives

The human health risk assessment was conducted for the products of the pathways most prone to plant and microbial transformation (Table 9). In the case of derivative D-17, the mutagenicity, rodent carcinogenicity (NTP and FDA datasets), and skin sensitization (weak vs. strong) exhibited improvement by reaching non-toxic levels, while the hepatotoxicity level remained unchanged fundamentally (the drug efficacy increased by 3.73%) and the rat oral toxicity level *LD*_50_ (g/kg) reduced by 29.97%. The developmental toxicity potential, skin irritation, skin sensitization (non vs. sens), and ocular irritancy exhibited the same toxicity level as earlier. These results indicated an overall significantly reduced human health risk for the modified QNs.

Furthermore, the hepatotoxicity levels of the intermediate and final products of NOR and its derivative D-17 in both plant and microbial transformation pathways remained unchanged prior to and after the modification, while the rodent carcinogenicity (NTP and FDA datasets) and mutagenicity reduced remarkably. The levels of DTP, skin irritancy, skin sensitization (non vs. sens; weak vs. strong), and ocular irritancy (non vs. irritant; mild vs. moderate/severe) remain unchanged. In addition, the hydroxylation reaction of piperazine rings could result in the transformation products having a certain degree of oral toxicity in rats, although the *LD*_50_ level remained unchanged. Therefore, it was inferred that the human health risk of the modified QNs was markedly reduced, and the toxicity and the human health risk of the transformation products of these molecules also decreased significantly.

## 4. Conclusions

In the present study, the fuzzy comprehensive evaluation method was used in combination with the weighted average method to construct a 3D-QSAR (CoMSIA) model for the plant–microbial degradation effect of QNs. The constructed model was then effectively applied for the molecular modification of environment-friendly QNs. Further, the simulation of plant and microbial degradation pathways of the QNs molecules was performed to assess the human health risks associated with these molecules and their transformation products after their molecular modification, which revealed that the potential risks had decreased significantly. A comprehensive system of model construction, molecular modification, drug environment-friendliness and functional properties evaluation, degradation pathway simulation, and drug human health risk assessment was successfully established in the present study, providing a solid theoretical basis for novel ideas and approaches for source control and drug design. However, there are still some limitations of this study, such as the calculation of binding energy did not consider the influence of external conditions in the process of model construction, or the designed derivatives were not synthesized experimentally. To solve the above problems, the present study constructed the model with molecular structural parameters as the main consideration, which have been proved to be reliable and accurate, and provided theoretical guidance for the design and synthesis of QNs. It is expected that the synthesis and experimental verification of QNs will be carried out in future work.

## Figures and Tables

**Table 1:** The plant, microbial, and the plant–microbial synergistic degradation effect values of QNs.

No.	Compounds	Abbreviations	$\mathrm{Binding}\;\mathrm{Energy}\;(\Delta G_{b},\;\mathrm{kcal}/\mathrm{mol})$				Synergistic Value (B, kcal/mol)
			1PA2	1MNP	1GYC	1B85	
1	Difloxacin	DIF	25.182	−132.832	−134.927	−154.835	−38.709
2	Enrofloxacin	ENR	−99.515	−113.013	−129.396	−150.755	−37.689
3	Norfloxacin	NOR	−54.030	−34.061	−78.238	−136.532	−34.133
4	Lomefloxacin	LOM	−86.227	−75.699	−137.530	−156.759	−39.190
5	Levofloxacin	LEV	−102.767	−108.691	−131.059	−88.252	−32.765
6	Pefloxacin	PEF	−113.216	−76.226	−122.837	−141.439	−35.360
7	Fleroxacin	FLE	−57.882	−118.085	−144.362	−148.723	−37.181
8	Ciprofloxacin	CIP	−74.823	−84.186	−126.139	−82.975	−31.535
9	Balofloxacin	BAL	−111.963	−113.730	−86.164	−137.961	−34.490
10	Marbofloxacin	MAR	−129.744	−81.674	−143.075	−135.558	−35.769
11	Pipemidic acid	PIP	−75.560	8.497	−116.738	−101.248	−29.185
12	Cinoxacin	CIN	−76.538	−108.556	−96.815	−86.754	−27.139
13	Enoxacin	ENO	−45.854	−38.068	−88.957	−114.112	−28.528
14	Danofloxacin	DAN	−83.938	−117.966	−115.184	−167.616	−41.904
15	Gatifloxacin	GAT	−68.580	−178.118	−111.547	−170.889	−44.530
16	Ofloxacin	OFL	−84.910	−107.253	−84.247	−51.343	−26.813
17	Rufloxacin	RUF	−136.757	−144.343	−121.198	−122.849	−36.086
18	Pazufloxacin	PAZ	−82.190	−11.624	−112.995	−103.151	−28.249
19	Nadifloxacin	NAD	−109.028	−80.519	−121.006	−116.732	−30.252
20	Moxifloxacin	MOX	−112.860	−192.333	−95.345	−170.045	−48.083
21	Sparfloxacin	SPA	−95.365	−102.209	−123.286	−132.893	−33.223
22	Sarafloxacin	SAR	−98.545	−125.849	−131.262	−124.028	−32.816
23	Amifloxacin	AMI	−120.565	−120.368	−148.711	−129.569	−37.178
24	Besifloxacin	BES	−121.613	−77.347	−122.906	−187.176	−46.794
25	Clinafloxacin	CLI	−97.981	−95.433	−121.897	−131.993	−32.998
26	Grepafloxacin	GRE	−126.186	−109.368	−98.129	−168.713	−42.178
27	Orbifloxacin	ORB	−96.297	−97.955	−136.773	−161.230	−40.308
28	Sitafloxacin	SIT	−85.799	−138.752	−129.410	−183.378	−45.845
29	Temafloxacin	TEM	−118.200	−57.170	−128.660	−206.845	−51.711

**Table 2:** Parameters of the CoMSIA model for the plant, microbial, and the plant–microbial synergistic degradation effects of QNs.

CoMSIA		q^2^	*n*	R^2^	(R^2^ − q^2^)/R^2^	SEE	F	Q^2^	cSDEP	dq^2^/dr^2^yy	r^2^_pred_
For plant–microbial	4 enzymes	0.707	10	1.000	29%	0.223	1816.658	0.571	8.009	1.664	0.764
For plant	1PA2	0.833	6	0.995	16%	0.640	230.436	0.705	5.050	1.599	0.854
For microbial	1MNP	0.695	6	0.997	30%	0.862	507.790	0.503	10.529	1.529	0.921
	1B85	0.707	10	0.999	29%	0.396	974.934	0.459	10.154	1.385	0.870
	1GYC	0.743	5	0.990	25%	0.558	198.713	0.501	3.907	2.116	0.678

**Table 3:** Substitution sites and groups in NOR.

No.	Compounds	Substitution Sites and Groups
3	NOR	
D-1	Derivative-1	13-Sulfydryl
D-2	Derivative-2	13-Chlorine
D-3	Derivative-3	13-Fluorine
D-4	Derivative-4	1- Bromine
D-5	Derivative-5	2- Fluorine
D-6	Derivative-6	2- Methyl
D-7	Derivative-7	2-Amidogen
D-8	Derivative-8	2-Sulfydryl
D-9	Derivative-9	2-Carboxyl
D-10	Derivative-10	2-Trifluoromethyl
D-11	Derivative-11	2-Fluoromethane
D-12	Derivative-12	13-Sulfydryl, 1-Bromine
D-13	Derivative-13	13-Sulfydryl, 2-Fluorine
D-14	Derivative-14	13-Sulfydryl, 2-Methyl
D-15	Derivative-15	13-Sulfydryl, 2-Amidogen
D-16	Derivative-16	13-Sulfydryl, 2-Sulfydryl
D-17	Derivative-17	13-Sulfydryl, 2-Carboxyl
D-18	Derivative-18	13-Sulfydryl, 2-Trifluoromethyl
D-19	Derivative-19	13-Sulfydryl, 2-Fluoromethane
D-20	Derivative-20	13-Chlorine, 1-Bromine
D-21	Derivative-21	13-Chlorine, 2-Fluorine
D-22	Derivative-22	13-Chlorine, 2-Methyl
D-23	Derivative-23	13-Chlorine, 2-Amidogen
D-24	Derivative-24	13-Chlorine, 2-Sulfydryl
D-25	Derivative-25	13-Chlorine, 2-Carboxyl
D-26	Derivative-26	13-Chlorine, 2-Trifluoromethyl
D-27	Derivative-27	13-Chlorine, 2-Fluoromethane
D-28	Derivative-28	13-Fluorine, 1-Bromine
D-29	Derivative-29	13-Fluorine, 2-Fluorine
D-30	Derivative-30	13-Fluorine, 2-Methyl
D-31	Derivative-31	13-Fluorine, 2-Amidogen
D-32	Derivative-32	13-Fluorine, 2-Sulfydryl
D-33	Derivative-33	13-Fluorine, 2-Carboxyl
D-34	Derivative-34	13-Fluorine, 2-Trifluoromethyl
D-35	Derivative-35	13-Fluorine, 2-Fluoromethane

**Table 4:** The values and change rates of the plant, microbial, and plant–microbial synergistic degradation effects of NOR and its derivatives predicted using the CoMSIA model.

No.	Synergistic Degradation Effect		Plant Degradation Effect		Microbial Degradation Effect					
			Peroxidase (1PA2)		Manganese Peroxidase (1MNP)		Lignin Peroxidase (1B85)		Laccase (1GYC)	
	Pred.	Change Rate (%)	Pred.	Change Rate (%)	Pred.	Change Rate (%)	Pred.	Change Rate (%)	Pred.	Change Rate (%)
NOR	−34.133		−13.508		−8.515		−34.133		−19.560	
D-1	−41.476	21.51	−22.395	65.80	−24.503	187.76	−38.547	12.93	−25.518	30.46
D-2	−40.962	20.01	−22.802	68.81	−26.370	209.69	−37.985	11.29	−25.736	31.57
D-3	−40.264	17.96	−22.431	66.06	−25.574	200.34	−36.847	7.95	−25.247	29.07
D-4	−37.647	10.30	−20.081	48.67	−22.109	159.65	−37.158	8.86	−21.794	11.42
D-5	−39.659	16.19	−21.930	62.35	−26.472	210.89	−41.139	20.53	−26.767	36.85
D-6	−37.730	10.54	−19.620	45.25	−21.055	147.27	−32.266	−5.47	−22.889	17.02
D-7	−38.463	12.69	−22.785	68.68	−22.665	166.18	−31.787	−6.87	−26.959	37.83
D-8	−38.084	11.58	−23.036	70.54	−22.719	166.81	−31.336	−8.19	−25.945	32.64
D-9	−37.642	10.28	−22.492	66.51	−22.367	162.68	−33.497	−1.86	−25.880	32.31
D-10	−37.863	10.93	−17.501	29.57	−20.936	145.87	−39.716	16.36	−21.092	7.83
D-11	−37.960	11.21	−18.353	35.87	−19.453	128.46	−31.118	−8.83	−22.320	14.11
D-12	−39.697	16.30	−19.601	45.11	−23.427	175.13	−39.766	16.50	−21.912	12.02
D-13	−40.974	20.04	−22.558	67.00	−24.818	191.46	−40.992	20.09	−26.805	37.04
D-14	−40.344	18.20	−20.468	51.53	−23.313	173.79	−36.819	7.87	−23.498	20.13
D-15	−41.174	20.63	−23.340	72.79	−24.561	188.44	−37.111	8.72	−27.152	38.81
D-16	−40.751	19.39	−23.709	75.52	−24.861	191.97	−36.279	6.29	−26.317	34.54
D-17	−39.598	16.01	−22.146	63.95	−23.723	178.60	−36.198	6.05	−26.126	33.57
D-18	−40.278	18.00	−17.726	31.23	−22.833	168.15	−43.327	26.94	−21.470	9.76
D-19	−39.670	16.22	−19.617	45.23	−22.965	169.70	−39.374	15.35	−22.482	14.94
D-20	−39.165	14.74	−19.715	45.96	−24.314	185.54	−39.254	15.00	−21.891	11.92
D-21	−40.431	18.45	−22.691	67.99	−26.170	207.34	−40.512	18.69	−26.815	37.09
D-22	−35.528	4.09	−23.541	74.28	−23.630	177.51	−41.617	21.93	−27.929	42.79
D-23	−40.614	18.99	−23.527	74.18	−25.955	204.82	−36.655	7.39	−27.258	39.36
D-24	−40.199	17.77	−23.860	76.64	−26.209	207.80	−35.791	4.86	−26.390	34.92
D-25	−39.083	14.50	−22.262	64.81	−24.560	188.43	−35.597	4.29	−26.084	33.35
D-26	−34.702	1.67	−21.147	56.56	−21.602	153.69	−48.717	42.73	−26.756	36.79
D-27	−34.582	1.32	−22.809	68.86	−22.980	169.88	−44.842	31.37	−27.215	39.14
D-28	−37.599	10.15	−20.159	49.24	−21.980	158.13	−38.281	10.84	−22.085	12.91
D-29	−39.758	16.48	−22.317	65.22	−25.357	197.79	−39.869	16.80	−26.477	35.36
D-30	−39.205	14.86	−20.095	48.77	−23.667	177.94	−35.861	5.06	−23.026	17.72
D-31	−36.572	7.15	−25.676	90.09	−24.773	190.93	−41.135	20.51	−30.032	53.54
D-32	−36.482	6.88	−26.681	97.53	−26.457	210.71	−40.807	19.55	−29.724	51.96
D-33	−38.599	13.08	−22.699	68.05	−28.217	231.38	−34.088	−0.13	−27.707	41.65
D-34	−39.239	14.96	−17.691	30.97	−23.286	173.47	−42.904	25.70	−21.280	8.79
D-35	−38.567	12.99	−19.225	42.33	−23.257	173.13	−38.399	12.50	−21.954	12.24

**Table 5:** Evaluation of the environment-friendliness of NOR and its derivatives.

No.	Bioaccumulation		Soil Adsorbability	
	log *K*_ow_	Change Rate (%)	log *K*_oc_	Change Rate (%)
3	−1.03		−0.392	
D-1	0.4	−138.83	0.399	−201.79%
D-2	−0.13	−87.38	0.105	−126.79%
D-3	−0.44	−57.28	−0.066	−83.16%
D-4	−0.45	−56.31	−0.072	−81.63%
D-5	−1.34	30.10	−0.542	38.27%
D-10	−0.38	−63.11	−0.033	−91.58%
D-12	0.25	−124.27	0.316	−180.61%
D-13	−0.64	−37.86	−0.155	−60.46%
D-14	−0.09	−91.26	0.128	−132.65%
D-15	−1.32	28.16	−0.531	35.46%
D-16	−0.66	−35.92	−0.166	−57.65%
D-17	−1.91	85.44	−0.857	118.62%
D-18	0.33	−132.04	0.36	−191.84%
D-19	−0.15	−85.44	0.094	−123.98%
D-20	−0.27	−73.79	0.028	−107.14%
D-21	−1.16	12.62	−0.442	12.76%
D-23	−1.85	79.61	−0.824	110.20%
D-24	−1.18	14.56	−0.454	15.82%
D-25	−2.44	136.89	−1.15	193.37%
D-28	−0.59	−42.72	−0.149	−61.99%
D-29	−1.48	43.69	−0.619	57.91%
D-30	−0.93	−9.71	−0.337	−14.03%
D-31	−2.16	109.71	−0.996	154.08%
D-33	−2.75	166.99	−1.322	237.24%
D-34	−0.51	−50.49	−0.105	−73.21%
D-35	−0.99	−3.88	−0.37	−5.61%

**Table 6:** Functional evaluation parameters of molecular stability and genotoxicity for NOR and its derivatives.

No.	Stability					Genotoxicity	
	Molecular Structure Stability			Molecular Metabolic Stability			
	Frequency (cm^−1^)	Total Energy (a.u.)	Change Rate (%)	Bayesian Score	Change Rate (%)	pLOEC	Change Rate (%)
3	24.82	−1109.899		−3.860		8.055	
D-5	28.90	−1130.418	1.85	−3.434	−11.05	7.559	−6.16
D-15	26.16	−1484.726	33.77	−2.516	−34.83	8.435	4.72
D-17	18.46	−1617.921	45.77	−2.214	−42.65	8.159	1.29
D-21	22.80	−1589.958	43.25	−2.876	−25.49	7.464	−7.34
D-23	23.88	−1546.137	39.30	−2.772	−28.18	7.97	−1.06
D-24	22.11	−1888.951	70.19	−4.553	17.96	7.814	−2.99
D-25	18.30	−1679.331	51.30	−2.471	−36.00	7.692	−4.51
D-29	27.90	−1229.614	10.79	−2.449	−36.54	7.311	−9.24
D-30	23.08	−1169.793	5.40	−3.051	−20.97	7.483	−7.10
D-31	27.64	−1185.797	6.84	−2.163	−43.96	7.766	−3.59
D-33	18.66	−1318.992	18.84	−2.044	−47.06	7.466	−7.31
D-35	13.05	−1268.980	14.33	−2.876	−25.50	7.443	−7.60

**Table 7:** Evaluation criteria for the functional evaluation parameters of stability and genotoxicity.

Property	Parameter	Value	Description	References
Stability of molecular structure	Frequency (cm^−1^)	>0	Stable	[108]
	Total Energy (a.u.)	Lower	Higher	[109]
Stability of molecular metabolism	Bayesian Score	<0.161	Non-inhibitor	[110]
		>0.161	Inhibitor	
Genotoxicity	pLOEC	Higher	Higher	[73]

**Table 8:** Calculation of the ΔE for the plant and microbial transformation pathways of NOR and its derivative D-17.

Transformation	Pathway	NOR				D-17				Change Rate (%)
		Reactant	Product	ΔE (kJ/mol)	ΔE (Total) (kJ/mol)	Reactant	Product	ΔE (kJ/mol)	ΔE (Total) (kJ/mol)	
Plant Degradation	Pathway A	NOR	N0-1	7.021	1042.390	D-17	D-17-1	0.098	569.287	↓* −98.61
		N0-1	N0-2	575.569		D-17-1	D-17-2	9.357		↓−98.37
		N0-2	N0-3	459.801		D-17-2	D-17-3	559.832		↑* 21.76
	Pathway B	NOR	N0-4	59.555	63.301	D-17	D-17-4	25.885	25.885	↓−56.54
		N0-4	N0-5	0.364		D-17-4	D-17-5	−5.233	-	-
		N0-5	N0-6	3.381		D-17-5	D-17-6	527.635		
Microbial Degradation	Pathway A	NOR	N0-1	59.555	59.555	D-17	D-17-1	25.885	25.885	↓−56.54
	Pathway B	NOR	N0-2	102.450	644.955	D-17	D-17-2	86.674	147.276	↓−15.40
		N0-2	N0-3	−39.949		D-17-2	D-17-3	−2.983		-
			N0-4	542.506			D-17-4	60.602		↓−88.83
	Pathway C	NOR	N0-5	14.263	38.950	D-17	D-17-5	57.831	68.585	↑305.47
		N0-5	N0-6	24.687		D-17-5	D-17-6	10.754		↓−56.44
	Pathway D	NOR	N0-7	40.871	63.897	D-17	D-17-7	−17.971	-	-
		N0-7	N0-8	1.274		D-17-7	D-17-8	646.370		
		N0-8	N0-9	21.752		D-17-8	D-17-9	343.622		

* “↓” indicates that the value decreases, and “↑” indicates that the value increases.

**Table 9:** Assessment of the human health risk raised by the plant and microbial transformation products of NOR prior to and after the modification based on pharmacokinetics and toxicokinetics.

Transformation	Pathway	Product	ADMET EXT Hepatotoxic (Non vs. Toxic)		Ames Mutagenicity (Non vs. Mutagenicity)	NTP Rodent Carcinogenicity (Non vs. Carcinogen)				FDA Rodent Carcinogenicity (Non vs. Carcinogen)			
			Hepatotoxicity	Change Rate (%)		Male Rat	Female Rat	Male Mouse	Female Mouse	Male Rat	Female Rat	Male Mouse	Female Mouse
		NOR	1.861/T		0.937/M	0.671/C	0.500/N	0.674/C	0.378/N	0.091/N	0.137/N	0.125/N	0.217/N
Plant Degradation	A	N0-1	1.131/T	−39.21	0.728/M	0.578/N	0.448/N	0.608/C	0.522/N	0.154/N	0.183/N	0.171/N	0.212/N
		N0-2	−0.338/N	−118.15	0.671/N	0.589/N	0.403/N	0.600/C	0.378/N	0.157/N	0.184/N	0.154/N	0.221/N
	B	N0-4	−0.106/N	−105.71	0.703/N	0.606/C	0.464/N	0.517/N	0.479/N	0.312/N	0.230/N	0.220/N	0.215/N
Microbial Degradation	A	N0-1	−0.106/N	−105.71	0.703/N	0.606/C	0.464/N	0.517/N	0.479/N	0.312/N	0.230/N	0.220/N	0.215/N
	B	N0-2	−1.040/N	−155.88	0.707/N	0.566/C	0.395/N	0.568/C	0.434/N	0.218/N	0.217/N	0.216/N	0.206/N
		N0-4	−0.277/N	−114.88	0.725/N	0.311/C	0.363/N	0.395/N	0.311/N	0.257/N	0.205/N	0.234/N	0.208/N
		D-17	1.931/T		0.663/N	0.591/N	0.445/N	0.486/N	0.362/N	0.171/N	0.205/N	0.140/N	0.213/N
Plant Degradation	A	D-17-1	1.593/T	−17.47	0.666/N	0.515/N	0.381/N	0.462/N	0.335/N	0.173/N	0.204/N	0.145/N	0.213/N
		D-17-2	−1.185/N	−161.36	0.632/N	0.532/N	0.332/N	0.418/N	0.277/N	0.166/N	0.219/N	0.167/N	0.206/N
	B	D-17-4	0.382//N	−80.19	0.721/N	0.625/C	0.487/N	0.540/N	0.461/N	0.317/N	0.226/N	0.219/N	0.219/N
Microbial Degradation	A	D-17-1	0.382/N	−80.19	0.721/N	0.625/C	0.487/N	0.540/N	0.461/N	0.317/N	0.226/N	0.219/N	0.219/N
	B	D-17-2	−1.280/N	−166.32	0.679/N	0.539/N	0.398/N	0.354/N	0.363/N	0.238/N	0.227/N	0.189/N	0.205/N
		D-17-4	0.307/N	−84.09	0.632/N	0.639/C	0.418/N	0.541/N	0.279/N	0.218/N	0.212/N	0.151/N	0.217/N
**Transformation**	**Pathway**	**Product**	**Rat Oral**	**Developmental Toxicity Potential (DTP) (Non vs. Toxic)**	**Skin Irritancy (Non vs. Irritant)**	**Skin Sensitization**				**Ocular Irritancy**			
			*LD*_50_^*^ (g/kg)			**Non vs. Sens**		**Weak vs. Strong**		**Non vs. Irritant**		**Mild vs. Moderate/Severe**	
		NOR	1.955/C4	0.707/T	0.957/N	0.800/S		0.897/S		0.999/I		0.861/M	
Plant Degradation	A	N0-1	4.166/C5	0.651/T	0.966/N	0.810/S		0.890/S		0.999/I		0.841/M	
		N0-2	1.622/C4	0.669/T	0.949/N	0.773/S		0.863/W		0.999/I		0.886/M	
	B	N0-4	0.278/C3	0.566/T	0.950/N	0.856/S		0.925/S		0.999/I		0.833/M	
Microbial Degradation	A	N0-1	0.278/C3	0.566/T	0.950/N	0.856/S		0.925/S		0.999/I		0.833/M	
	B	N0-2	2.731/C5	0.625/T	0.962/N	0.788/S		0.889/S		0.999/I		0.867/M	
		N0-4	1.528/C4	0.541/T	0.906/N	0.861/S		0.913/S		0.999/I		0.828/M	
		D-17	1.369/C4	0.631/T	0.961/N	0.786/S		0.850/W		0.999/I		0.844/M	
Plant Degradation	A	D-17-1	0.643/C4	0.637/T	0.959/N	0.774/S		0.848/W		0.999/I		0.857/M	
		D-17-2	0.531/C4	0.644/T	0.952/N	0.748/S		0.795/W		0.999/I		0.871/M	
	B	D-17-4	0.236/C3	0.547/T	0.949/N	0.844/S		0.976/S		0.999/I		0.836/M	
Microbial Degradation	A	D-17-1	0.236/C3	0.547/T	0.949/N	0.844/S		0.976/S		0.999/I		0.836/M	
	B	D-17-2	1.127/C4	0.599/T	0.967/N	0.775/S		0.858/W		0.999/I		0.844/M	
		D-17-4	1.116/C4	0.625/T	0.966/N	0.772/S		0.860/W		0.999/I		0.862/M	

^*^ Assessment based on “Acute toxicity estimate (ATE) values and criteria for acute toxicity hazard categories” (Table 3.1.1), cited from [117] Boatman, R.; Kelsey, J.; Ball, N. Acute toxicity classification for ethylene glycol mono-n-butyl ether under the Globally Harmonized System. *Regul. Toxicol. Pharmacol.* **2014**, *68*, 41–50.

## Data Availability

Not applicable.

## References

[1] He X.T., Deng M.C., Wang Q., Yang Y.T., Yang Y.F., Nie X.P. (2016). Residues and health risk assessment of quinolones and sulfonamides in cultured fish from Pearl River Delta, China. Aquaculture.

[2] Xu W.H., Zhang G., Zou S.C., Li X., Liu Y.C. (2007). Determination of selected antibiotics in the Victoria Harbour and the Pearl River, South China using high-performance liquid chromatography-electrospray ionization tandem mass spectrometry. Environ. Pollut..

[3] Andreozzi R., Raffaele M., Nicklas P. (2003). Pharmaceuticals in STP effluents and their solar photodegradation in aquatic environment. Chemosphere.

[4] Doorslaer V.X., Dewulf J., Langenhove V.H., Demeestere K. (2014). Fluoroquinolone antibiotics: An emerging class of environmental micropollutants. Sci. Total Environ..

[5] Xie W.Y., McGrath P.S., Su J.Q., Hirsch R.P., Clark M.I., Shen Q.R., Zhu Y.G., Zhao F.J. (2016). Long-Term Impact of Field Applications of Sewage Sludge on Soil Antibiotic Resistome. Environ. Sci. Technol..

[6] Donkor S.E., Newman J.M., Tay C.K.S., Dayie T.K.D.N., Bannerman E., Olu-Taiwo M. (2011). Investigation into the risk of exposure to antibiotic residues contaminating meat and egg in Ghana. Food Control..

[7] Al-Ahmad A., Daschner F.D., Kümmerer K. (1999). Biodegradability of Cefotiam, Ciprofloxacin, Meropenem, Penicillin G, and Sulfamethoxazole and Inhibition of Waste Water Bacteria. Arch. Environ. Contam. Toxicol..

[8] Li B., Zhang T. (2010). Biodegradation and adsorption of antibiotics in the activated sludge process. Environ. Sci. Technol..

[9] Zhou L.J., Ying G.G., Liu S., Zhao J.L., Yang B., Chen Z.F., Lai H.J. (2013). Occurrence and fate of eleven classes of antibiotics in two typical wastewater treatment plants in South China. Sci. Total Environ..

[10] Sevcan A. (2016). Enhanced biodegradation of antibiotic combinations via the sequential treatment of the sludge resulting from pharmaceutical wastewater treatment using white-rot fungi *Trametes versicolor* and *Bjerkandera adusta*. Appl. Microbiol. Biotechnol..

[11] Bergheim M., Gminski R., Spangenberg B., Debiak M., Bürkle A., Mersch-Sundermann V., Kümmerer K., Gieré R. (2015). Antibiotics and sweeteners in the aquatic environment: Biodegradability, formation of phototransformation products, and in vitro toxicity. Environ. Sci. Pollut. Res. Int..

[12] Golet M.E., Xifra I., Siegrist H., Alder C.A., Giger W. (2003). Environmental exposure assessment of fluoroquinolone antibacterial agents from sewage to soil. Environ. Sci. Technol..

[13] Dorival-García N., Zafra-Gómez A., Navalón A., González-López J., Hontoria E., Vílchez J.L. (2013). Removal and degradation characteristics of quinolone antibiotics in laboratory-scale activated sludge reactors under aerobic, nitrifying and anoxic conditions. J. Environ. Manag..

[14] Alexy R., Kümpel T., Kümmerer K. (2004). Assessment of degradation of 18 antibiotics in the Closed Bottle Test. Chemosphere.

[15] Senta I., Terzic S., Ahel M. (2013). Occurrence and fate of dissolved and particulate antimicrobials in municipal wastewater treatment. Water Res..

[16] Rodriguez-Mozaz S., Chamorro S., Marti E., Huerta B., Gros M., Sànchez-Melsió A., Borrego M.C., Barceló D., Balcázar L.J. (2015). Occurrence of antibiotics and antibiotic resistance genes in hospital and urban wastewaters and their impact on the receiving river. Water Res..

[17] Xiong J.Q., Kurade B.M., Patil V.D., Jang M., Paeng K.J., Jeon B.H. (2017). Biodegradation and metabolic fate of levofloxacin via a freshwater green alga, Scenedesmus obliquus in synthetic saline wastewater. Algal Res..

[18] Zhang X., Zhao H.X., Du J., Qu Y.X., Shen C., Tan F., Chen J.W., Quan X. (2017). Occurrence, removal, and risk assessment of antibiotics in 12 wastewater treatment plants from Dalian, China. Environ. Sci. Pollut. Res. Int..

[19] Tolls J. (2001). Sorption of veterinary pharmaceuticals in soils: A review. Environ. Sci. Technol..

[20] Boxall B.A.A., Johnson P., Smith J.E., Sinclair J.C., Stutt E., Levy S.L. (2006). Uptake of veterinary medicines from soils into plants. J. Agric. Food Chem..

[21] Chen Y., Rosazza J.P.N., Reese C.P., Chang H.Y., Nowakowski M.A., Kiplinger J.P. (1997). Microbial models of soil metabolism: Biotransformations of danofloxacin. J. Ind. Microbiol. and Biotechnol..

[22] Ma B., He Y., Chen H.H., Xu J.M., Rengel Z. (2010). Dissipation of polycyclic aromatic hydrocarbons (PAHs) in the rhizosphere: Synthesis through meta-analysis. Environ. Pollut..

[23] Riley D., Barber S.A. (1971). Effect of Ammonium and Nitrate Fertilization on Phosphorus Uptake as Related to Root-Induced pH Changes at the Root-Soil Interface. Soil Sci. Soc. Am. J..

[24] Gahoonia T.S., Claassen N., Jungk A. (1992). Mobilization of phosphate in different soils by ryegrass supplied with ammonium or nitrate. Plant. Soil.

[25] Mackova M., Prouzova P., Stursa P., Ryslava E., Uhlik O., Beranova K., Rezek J., Kurzawova V., Demnerova K., Macek T. (2009). Phyto/rhizoremediation studies using long-term PCB-contaminated soil. Environ. Sci. Pollut. Res..

[26] Moritsuka N., Yanai J., Kosaki T. (2012). Effect of plant growth on the distribution and forms of soil nutrients in the rhizosphere. Soil Sci. Plant. Nutr..

[27] Tsednee M., Mak Y.W., Chen Y.R., Yeh K.C. (2012). A sensitive LC-ESI-Q-TOF-MS method reveals novel phytosiderophores and phytosiderophore-iron complexes in barley. N. Phytol..

[28] Chekol T., Vough R.L., Chaney L.R. (2004). Phytoremediation of polychlorinated biphenyl-contaminated soils: The rhizosphere effect. Environ. Int..

[29] Gilbert E.S., Crowley D.E. (1998). Repeated application of carvone-induced bacteria to enhance biodegradation of polychlorinated biphenyls in soil. Appl. Microbiol. Biotechnol..

[30] Yi H., Crowley E.D. (2007). Biostimulation of PAH Degradation with Plants Containing High Concentrations of Linoleic Acid. Environ. Sci. Technol..

[31] Rasmussen K.S. (1990). Molecular and physiological aspects of plant peroxidases. Plant. Mol. Biol. Rep..

[32] Zhao H.M., Huang H.B., Du H., Lin J., Xiang L., Li Y.W., Cai Q.Y., Li H., Mo C.H., Liu J.S. (2018). Intraspecific variability of ciprofloxacin accumulation, tolerance, and metabolism in Chinese flowering cabbage (*Brassica parachinensis*). J. Hazard. Mater..

[33] Olga K., Oksana G., Radka K., Miroslav F., Roman G. (2017). Antibiotics degradation in soil: A case of clindamycin, trimethoprim, sulfamethoxazole and their transformation products. Environ. Pollut..

[34] Marco-Urrea E., Pérez-Trujillo M., Vicent T., Caminal G. (2009). Ability of white-rot fungi to remove selected pharmaceuticals and identification of degradation products of ibuprofen by *Trametes versicolor*. Chemosphere.

[35] Prieto A., Möder M., Rodil R., Adrian L., Marco-Urrea E. (2011). Degradation of the antibiotics norfloxacin and ciprofloxacin by a white-rot fungus and identification of degradation products. Bioresour. Technol..

[36] Asgher M., Bhatti H.N., Ashraf M., Legge L.R. (2008). Recent developments in biodegradation of industrial pollutants by white rot fungi and their enzyme system. Biodegradation.

[37] Liu H. (2019). Study on the Degradation of Sulfamerazine by Horseradish Peroxidase. Master’s Thesis.

[38] Kurnik K., Treder K., Twarużek M., Grajewski J., Tretyn A., Tyburski J. (2018). Potato Pulp as the Peroxidase Source for 2,4-Dichlorophenol Removal. Waste Biomass Valoriz..

[39] Čvančarová M., Moeder M., Filipová A., Cajthaml T. (2015). Biotransformation of fluoroquinolone antibiotics by ligninolytic fungi—Metabolites, enzymes and residual antibacterial activity. Chemosphere.

[40] Gao Y.H., Jia L.H., Wu Z.J., Sun Z.J. (2012). Effect of albendazole on microstructure and ultrastructure during spermiogenesis in earthworms. Acta Sci. Circumst..

[41] Piotrowicz-Cieślak I.A., Adomas B., Nałęcz-Jawecki G., Michalczyk J.D. (2010). Phytotoxicity of Sulfamethazine Soil Pollutant to Six Legume Plant Species. J. Toxicol. Environ. Health.

[42] D’Abrosca B., Fiorentino A., Izzo A., Cefarelli G., Pascarella T.M., Uzzo P., Monaco P. (2008). Phytotoxicity evaluation of five pharmaceutical pollutants detected in surface water on germination and growth of cultivated and spontaneous plants. J. Environ. Sci. Health.

[43] Pruden A., Pei R.T., Heather S., Kenneth H.C. (2006). Antibiotic resistance genes as emerging contaminants: Studies in northern Colorado. Environ. Sci. Technol..

[44] Davison J. (1999). Genetic Exchange between Bacteria in the Environment. Plasmid.

[45] Allen K.H., Donato J., Wang H.H., Cloud-Hansen A.K., Davies J., Handelsman J. (2010). Call of the wild: Antibiotic resistance genes in natural environments. Nat. Rev. Microbiol..

[46] Uddin M., Chen J.W., Qiao X.L., Tian R., Arafat Y., Yang X.J. (2019). Bacterial community variations in paddy soils induced by application of veterinary antibiotics in plant-soil systems. Ecotoxicol. Environ. Saf..

[47] Jin C.X., Chen Q.Y., Sun R.L., Zhou Q.X., Liu J.J. (2009). Eco-toxic effects of sulfadiazine sodium, sulfamonomethoxine sodium and enrofloxacin on wheat, Chinese cabbage and tomato. Ecotoxicology.

[48] Azanu D., Styrishave B., Darko G., Weisser J.J., Abaidoo C.R. (2018). Occurrence and risk assessment of antibiotics in water and lettuce in Ghana. Sci. Total. Environ..

[49] Gill H.J., Hough S.J., Naisbitt D.J., Maggs J.L., Kitteringham N.R., Pirmohamed M., Park B.K. (1997). The relationship between the disposition and immunogenicity of sulfamethoxazole in the rat. J. Pharmacol. Exp. Ther..

[50] Fu Q.G., Zhang J.B., Borchardt D., Schlenk D., Gan J. (2017). Direct Conjugation of Emerging Contaminants in Arabidopsis: Indication for an Overlooked Risk in Plants?. Environ. Sci. Technol..

[51] Prosser S.R., Sibley K.P. (2015). Corrigendum to: “Human health risk assessment of pharmaceuticals and personal care products in plant tissue due to biosolids and manure amendments, and wastewater irrigation” [Environ. Int. 2015, 75, 223–233]. Environ. Int..

[52] Yana R., Jose M.D., Robert L., Joan S., Bi C.X., Charmi B., Chen L., Alexander S.R., Sebastian B., Stephen K.B. (2021). RCSB Protein Data Bank: Architectural Advances Towards Integrated Searching and Efficient Access to Macromolecular Structure Data from the PDB Archive. J. Mol. Biol..

[53] Østergaard L., Teilum K., Mirza O., Mattsson O., Petersen M., Welinder G.K., Mundy J., Gajhede M., Henriksen A. (2000). Arabidopsis ATP A_2_ peroxidase. Expression and high-resolution structure of a plant peroxidase with implications for lignification. Plant. Mol. Biol..

[54] Munirathinam S., Katsuyuki K., Michael H.G., Thomas L.P. (1994). Preliminary Crystallographic Analysis of Manganese Peroxidase from *Phanerochaete chrysosporium*. Med. Chem..

[55] Blodig W., Smith A.T., Doyle W.A., Piontek K. (2001). Crystal structures of pristine and oxidatively processed lignin peroxidase expressed in Escherichia coli and of the W171F variant that eliminates the redox active tryptophan 171. Implications for the reaction mechanism. J. Mol. Biol..

[56] Piontek K., Antorini M., Choinowski T. (2002). Crystal structure of a laccase from the fungus *Trametes versicolor* at 1.90-A resolution containing a full complement of coppers. J. Biol. Chem..

[57] Krovat E.M., Steindl T., Langer. T. (2004). Recent Advances in Docking and Scoring. Curr. Comput.-Aided Drug Des..

[58] Meng X.Y., Zhang H.X., Mezei M., Cui M. (2011). Molecular Docking: A Powerful Approach for Structure-Based Drug Discovery.. Curr. Comput.-Aided Drug Des..

[59] Hou Y.L., Zhao Y.Y., Li Y. (2020). Environmentally Friendly Fluoroquinolone Derivatives with Lower Plasma Protein Binding Rate Designed Using 3D-QSAR, Molecular Docking and Molecular Dynamics Simulation. Int. J. Environ. Res. Public Health.

[60] Gu W.W., Li Q., Li Y. (2020). Law and mechanism analysis of biodegradability of polychlorinated naphthalenes based on principal component analysis, QSAR models, molecular docking and molecular dynamics simulation. Chemosphere.

[61] Ogrizek M., Turk S., Lešnik S., Sosič I., Hodošček M., Mirković B., Kos J., Janežič D., Gobec S., Konc J. (2015). Molecular dynamics to enhance structure-based virtual screening on cathepsin B. J. Comput.-Aided Mol. Des..

[62] Childers M.C., Daggett V. (2017). Insights from molecular dynamics simulations for computational protein design. Mol. Syst. Des. Eng..

[63] Bessonov K., Vassall K.A., Harauz G. (2013). Parameterization of the proline analogue Aze (azetidine-2-carboxylic acid) for molecular dynamics simulations and evaluation of its effect on homo-pentapeptide conformations. J. Mol. Graphics Modell..

[64] Joo J.C., Pack S.P., Kim Y.H., Yoo Y.J. (2011). Thermostabilization of Bacillus circulans xylanase: Computational optimization of unstable residues based on thermal fluctuation analysis. J. Biotechnol..

[65] Wang C.H., Nguyen H.P., Pham K., Huynh D., Le N.T.B., Wang H.L., Ren P.Y., Luo R. (2016). Calculating protein-ligand binding affinities with MMPBSA: Method and error analysis. J. Comput. Chem..

[66] Wang P.Z. (1983). Fuzzy Set Theory and its Application.

[67] Jiang J.S., Wu Y., Chen F., Qian K. (2020). Application of analytic hierarchy process and fuzzy comprehensive evaluation method in graduate students’ academic evaluation. J. Shangqiu Norm. Univ..

[68] Gu W.W., Zhao Y.Y., Li Q., Li Y. (2019). Environmentally friendly polychlorinated naphthalenes (PCNs) derivatives designed using 3D-QSAR and screened using molecular docking, density functional theory and health-based risk assessment. J. Hazard. Mater..

[69] Sree G.V., Bathula C., Youi H.K., Kim H.S., Inn S.J., Im H. (2021). Photophysical and DFT investigation of imidazole-based hole transporting materials for phosphorescent OLEDs with high current efficiency. J. Mol. Liq..

[70] Furer V.L., Vandyukov A.E., Kleshnina S.R., Solovieva S.E., Antipin I.S., Kovalenko V.I. (2021). DFT study of conformation, hydrogen bonds, IR, and Raman spectra of the sodium salt of p-hexasulfonatocalix[6]arene DFT. J. Mol. Struct..

[71] Zhao X.H., Zhao Y.Y., Ren Z.X., Li Y. (2019). Combined QSAR/QSPR and molecular docking study on fluoroquinolones to reduce biological enrichment. Comput. Biol. Chem..

[72] Xiao T., Wei X.Y., Gao X.X., Gu Q., Wang L., Fang H., Gu L.Q., Qi H., Yi Y.Q. (2011). The pharmacokinetics of gigantol and syringic acid. Northwest. Pharm. J..

[73] Zhao X.H., Wang X.L., Li Y. (2019). Combined HQSAR method and molecular docking study on genotoxicity mechanism of quinolones with higher genotoxicity. Environ. Sci. Pollut. Res..

[74] Tian R. (2019). Uptake and Metabolism of Four Typical Antibiotics in Leafy Vegetables. Master’s Thesis.

[75] Wetzstein H.G., Schmeer N., Karl W. (1998). Degradation of the fluoroquinolone enrofloxacin by the brown rot fungus *Gloeophyllum striatum*: Identification of metabolites. Appl. Environ. Microbiol..

[76] Salahinejad M., Ghasemi J.B. (2014). 3D-QSAR studies on the toxicity of substituted benzenes to *Tetrahymena pyriformis*: CoMFA, CoMSIA and VolSurf approaches. Ecotoxicol. Environ. Saf..

[77] Liu S.C., Sun S.J., Cui P., Ding Y.F. (2019). Molecular Modification of Fluoroquinolone-Biodegrading Enzymes Based on Molecular Docking and Homology Modelling. Int. J. Environ. Res. Public Health.

[78] Gao Y.T. (2020). Quantitative structure-activity relationships studying the toxicity of metal and metal oxide nanoparticles and amphetamines. Master’s Thesis.

[79] Nath A., Kumer A., Zaben F., Khan M.W. (2021). Investigating the binding affinity, molecular dynamics, and ADMET properties of 2,3-dihydrobenzofuran derivatives as an inhibitor of fungi, bacteria, and virus protein. Beni-Suef Univ. J. Basic Appl. Sci..

[80] Kuthyala S., Hanumanthappa M., Kumar S.M., Sheik S., Karikannar N.G., Prabhu A. (2019). Crystal, Hirshfeld, ADMET, drug-like and anticancer study of some newly synthesized imidazopyridine containing pyrazoline derivatives. J. Mol. Struct..

[81] Liu Y. (2012). The research on the application of three kinds of QSAR softwares in the ecological classification of chemicals management. Master’s Thesis.

[82] Qu R.J., Liu H.X., Feng M.B., Yang X., Wang Z.Y. (2012). Investigation on Intramolecular Hydrogen Bond and Some Thermodynamic Properties of Polyhydroxylated Anthraquinones. J. Chem. Eng. Data.

[83] Yang L.Z., Liu M. (2019). 3D-QSAR Model of Polybrominated Biphenyls Tri-effect Modified by Standard Deviation Standardization Method and Its Application in Environmental-Friendly Molecular Modification. Chem. J. Chin. Univ..

[84] Veerasamy R., Rajak H., Jain A., Sivadasan S., Varghese P.C., Agrawal K.R. (2011). Validation of QSAR models—Strategies and importance. Int. J. Drug Des. Discov..

[85] Wang X.L., Gu W.W., Guo E.M., Cui C.Y., Li Y. (2017). Assessment of long-range transport potential of polychlorinated Naphthalenes based on three-dimensional QSAR models. Environ. Sci. Pollut. Res..

[86] Gu W.W., Chen Y., Li Y. (2017). Attenuation of the Atmospheric Migration Ability of Polychlorinated Naphthalenes (PCN-2) Based on Three-dimensional QSAR Models with Full Factor Experimental Design. Bull. Environ. Contam. and Toxicol..

[87] Yang L.Z., Liu M. (2020). A double-activity (green algae toxicity and bacterial genotoxicity) 3D-QSAR model based on the comprehensive index method and its application in fluoroquinolones’ modification. Int. J. Environ. Res. Public Health.

[88] Kasperkiewicz A., Pawliszyn J. (2020). Multiresidue pesticide quantitation in multiple fruit matrices via automated coated blade spray and liquid chromatography coupled to triple quadrupole mass spectrometry. Food Chem..

[89] Gu W.W., Chen Y., Zhang L., Li Y. (2016). Prediction of octanol-water partition coefficient for polychlorinated naphthalenes through three-dimensional QSAR models. Hum. Ecol. Risk Assess..

[90] Jazaeri S., Bock E.J., Bagagli P.M., Iametti S., Bonomi F., Seetharaman K. (2015). Structural modifications of gluten proteins in strong and weak wheat dough during mixing. Cereal Chem..

[91] Hu Y.M., Wang L.J., Li Z.G. (2017). Modification of protein structure and dough rheological properties of wheat flour through superheated steam treatment. J. Cereal Sci..

[92] Carrasquillo J.A., Bruland L.G., MacKay A.A., Vasudevan D. (2008). Sorption of ciprofloxacin and oxytetracycline zwitterions to soils and soil minerals: Influence of compound structure. Environ. Sci. Technol..

[93] Huijbers M.C.P., Flach C.F., Larsson D.G.J. (2019). A conceptual framework for the environmental surveillance of antibiotics and antibiotic resistance. Environ. Int..

[94] Sun H.Y., Shi X., Mao J.D., Zhu D.Q. (2010). Tetracycline sorption to coal and soil humic acids: An examination of humic structural heterogeneity. Environ. Toxicol. Chem..

[95] Heuer H., Smalla K. (2007). Manure and sulfadiazine synergistically increased bacterial antibiotic resistance in soil over at least two months. Environ. Microbiol..

[96] Fang H., Han L.X., Zhang H.P., Deng Y.F., Ge Q.Q., Mei J.J., Long Z.N., Yu Y.L. (2018). Repeated treatments of ciprofloxacin and kresoxim-methyl alter their dissipation rates, biological function and increase antibiotic resistance in manured soil. Sci. Total. Environ..

[97] Chung H.S., Lee Y.J., Rahman M.M., Abd El-Aty A.M., Lee H.S., Kabir M.H., Kim S.W., Park B.J., Kim J.E., Hacımüftüoğlu F. (2017). Uptake of the veterinary antibiotics chlortetracycline, enrofloxacin, and sulphathiazole from soil by radish. Sci. Total Environ..

[98] Migliore L., Cozzolino S., Fiori M. (2003). Phytotoxicity to and uptake of enrofloxacin in crop plants. Chemosphere.

[99] Žižek S., Zidar P. (2013). Toxicity of the ionophore antibiotic lasalocid to soil-dwelling invertebrates: Avoidance tests in comparison to classic sublethal tests. Chemosphere.

[100] Zhu D., An X.L., Chen Q.L., Yang X.R., Christie P., Ke X., Wu L.H., Zhu Y.G. (2018). Antibiotics Disturb the Microbiome and Increase the Incidence of Resistance Genes in the Gut of a Common Soil Collembolan. Environ. Sci. Technol..

[101] Pan M., Chu L.M. (2016). Adsorption and degradation of five selected antibiotics in agricultural soil. Sci. Total. Environ..

[102] Schafhauser B.H., Kristofco A.L., de Oliveira C.M.R., Brooks W.B. (2018). Global review and analysis of erythromycin in the environment: Occurrence, bioaccumulation and antibiotic resistance hazards. Environ. Pollut..

[103] Michelini L., Reichel R., Werner W., Ghisi R., Thiele-Bruhn S. (2012). Sulfadiazine Uptake and Effects on *Salix fragilis* L. and *Zea mays* L. Plants. Water Air Soil Pollut..

[104] Han Y., Zhou W.S., Tang Y., Shi W., Shao Y.Q., Ren P., Zhang J.M., Xiao G.Q., Sun H.X., Liu G.X. (2021). Microplastics aggravate the bioaccumulation of three veterinary antibiotics in the thick shell mussel *Mytilus coruscus* and induce synergistic immunotoxic effects. Sci. Total Environ..

[105] Li X.X., Zhang B.Y., Huang W., Cantwell C., Chen B. (2020). Integration of Fuzzy Matter-Element Method and 3D-QSAR Model for Generation of Environmentally Friendly Quinolone Derivatives. Int. J. Environ. Res. Public Health.

[106] Zhang W.H., Gu W.W., Sun R.H., Zhou M.Y., Han Z.Z., Li Y. (2021). An adjusted 3D-QSAR model for the combined activity of fluoroquinolones photodegradation and microbial degradation assisted by dynamic simulation and its application in molecular modification. Ecotoxicol. Environ. Saf..

[107] Zhang W.H., Sun R.H., Zhao X.H., Li Y. (2020). Environmental Conversion Path Inference of New Designed Fluoroquinolones and Their Potential Environmental Risk. Arch. Environ. Contam. Toxicol..

[108] Qiu Y.L., Jiang L., Li Y. (2018). Theoretical support for the enhancement of infrared spectrum signals by derivatization of phthalic acid esters using a pharmacophore model. Spectrosc. Lett..

[109] Brzozowski A.M., Pike A.C., Dauter Z., Hubbard R.E., Bonn T., Engström O., Ohman L., Greene G.L., Gustafsson J.A., Carlquist M. (1997). Molecular basis of agonism and antagonism in the oestrogen receptor. Nature.

[110] Sarra M., Khedidja B., Mohamed Y. (2021). Identification of 3-methoxycarpachromene and masticadienonic acid as new target inhibitors against trypanothione reductase from leishmania infantum using molecular docking and ADMET prediction. Molecules.

[111] Sandermann H. (1992). Plant-metabolism of xenobiotics. Trends Biochem. Sci..

[112] Farkas M.H., Berry J.O., Aga D.S. (2007). Chlortetracycline detoxification in maize via induction of glutathione S-transferases after antibiotic exposure. Environ. Sci. Technol..

[113] Goldstein M., Shenker M., Chefetz B. (2014). Insights into the uptake processes of wastewater-borne pharmaceuticals by vegetables. Environ. Sci. Technol..

[114] Malchi T., Maor Y., Tadmor G., Shenker M., Chefetz B. (2014). Irrigation of root vegetables with treated wastewater: Evaluating uptake of pharmaceuticals and the associated human health risks. Environ. Sci. Technol..

[115] Miller E.L., Nason S.L., Karthikeyan K.G., Pedersen J.A. (2016). Root uptake of pharmaceuticals and personal care product ingredients. Environ. Sci. Technol..

[116] Pan M., Chu L.M. (2017). Fate of antibiotics in soil and their uptake by edible crops. Sci. Total Environ..

[117] Boatman R., Kelsey J., Ball N. (2014). Acute toxicity classification for ethylene glycol mono-n-butyl ether under the Globally Harmonized System. Regul. Toxicol. Pharmacol..

[118] Huang J.J., Hu H.Y., Tang F., Li Y., Lu S.Q., Lu Y. (2011). Inactivation and reactivation of antibiotic-resistant bacteria by chlorination in secondary effluents of a municipal wastewater treatment plant. Water Res..

[119] Xia X.Q., Huang C.H., Xi B.D., Tan W.B., Tang Z.R. (2019). Review on biotransformation and mechanism of fluoroquinolone antibiotics from livestock manure. J. Agro-Environ. Sci..

[120] Jia Y., Khanal S.K., Shu H., Zhang H.Q., Chen G.H., Lu H. (2018). Ciprofloxacin degradation in anaerobic sulfate-reducing bacteria (SRB) sludge system: Mechanism and pathways. Water Res..

[121] Sun Y.H. (2015). Research on the Oxidation Mechanisms of Typical Organic Pollutants in the Atmosphere. Ph.D. Thesis.

